# Design of natural asphalt sulfamic acid (NA-NHSO_3_H) as a scalable natural asphalt-derived heterogeneous Brønsted acid to catalyze multicomponent reactions meeting green chemistry goals

**DOI:** 10.1016/j.heliyon.2024.e41492

**Published:** 2024-12-25

**Authors:** Sahar Abdolahi, Mohammad Soleiman-Beigi

**Affiliations:** Department of Chemistry, Faculty of Basic Sciences, Ilam University, P.O. Box 69315516, Ilam, Iran

**Keywords:** Green chemistry, Natural asphalt, Solid Brønsted acid catalysts, NA-NHSO_3_H, Polyhydroquinolines, tetrahydrobenzo[b]pyran, 3,4-Dihydropyrimidine-2(1H)-One/thiones

## Abstract

This study highlights an innovative approach to catalysis by utilizing natural asphalt as a support material for developing carbon-based catalysts. By leveraging the principles of green chemistry, the research aims to create recyclable and environmentally friendly heterogeneous catalytic systems. This aligns with the growing demand for greener technologies and the use of biocompatible materials in chemical processes. Natural asphalt (NA), being a sustainable resource, can potentially reduce the environmental impact associated with traditional catalytic materials. The development of Brønsted acid catalysts on this platform could enhance catalytic efficiency while promoting sustainability. By focusing on recyclable systems, the study also addresses the issue of waste generation in catalytic processes, further advancing the goals of green chemistry. Herein, highlights the emerging role of solid Brønsted acid catalysts (SBACs) in organic synthesis, focusing on a new catalyst known as NA-NHSO_3_H, which is based on natural asphalt. The synthesis of NA-NHSO_3_H is straightforward, and its characterization is comprehensively conducted using various analytical techniques such as Fourier transform-infrared spectroscopy (FT-IR), Scanning electron microscopy (SEM), Energy-dispersive X-ray -Map (EDX-Map), Brunauer-Emmett-Teller (BET), Thermogravimetric analysis (TGA) and Transmission electron microscopy (TEM). The catalyst demonstrates high efficacy in the synthesis of a range of heterocyclic compounds, showcasing yields that vary from good to excellent even under environmentally friendly conditions. One of the significant advantages of NA-NHSO_3_H is its easy separation and reusability, making it economical and aligned with green chemistry principles. Furthermore, the study mentions the practical applicability of this catalyst, evidenced by tests confirming its robustness in gram-scale reactions and through hot filtration tests.

## Highlights

• Its availability, cost-effectiveness, and carbon-rich composition make natural asphalt an attractive support.
• Natural asphalt can be a promising alternative for synthesizing solid acid catalysts.
• The NA-NHSO_3_H catalyst was used for multicomponent reactions in water-green solvent at room temperature.
• The high activity of the catalyst was carried out on a large scale for the synthesis of heterocyclic compounds.
• Our protocol had the advantages of low cost, excellent yield, recyclability and environmental benignity.

## Graphical abstract

**Figure:**
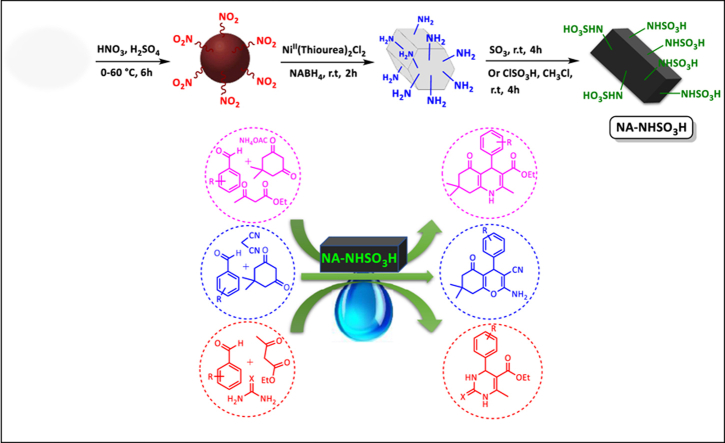

## 1 Introduction

Green chemistry represents a transformative approach in the chemical industry, emphasizing the minimization of hazardous substances, waste reduction, and energy efficiency [1]. Ongoing research and innovation in the field of green chemistry are crucial for developing new, sustainable technologies that can replace traditional processes and products. This includes the development of new catalysts, synthesis methods, and processes that align with the principles of green chemistry [[2], [3], [4]]. Heterogeneous Brønsted acids, which are solid acid catalysts, have gained significant attention in recent years due to their potential to replace traditional liquid acids in various chemical reactions. These solid acid catalysts offer several advantages over their liquid counterparts, making them a promising alternative for environmentally friendly and sustainable chemistry. They offer several advantages over traditional liquid acids, including reusability, reduced waste, and improved safety. Heterogeneous Brønsted acids are making significant contributions to advancing green chemistry by providing more sustainable and efficient alternatives to traditional liquid acid catalysts. Their solid form not only minimizes waste and reduces safety risks but also enhances operational practicality across various chemical processes. Continued research and innovation in this field can lead to the development of more efficient, safe, and sustainable practices across various industries, aligning with global goals for environmental conservation and sustainable development [5].

Brønsted acids such as HCl (hydrochloric acid), HNO_3_ (nitric acid), H_3_PO_4_ (phosphoric acid) and H_2_SO_4_ (sulfuric acid) play a very important role in promoting and improving catalytic performance. Despite the use of these homogeneous liquid acids as catalysts, they suffer from high corrosivity and toxicity, high quenching and regeneration, and noxious byproducts. The development of solid Brønsted acid catalysts (SBACs) marks an important advancement in the field of catalysis, reflecting a move towards greener and more efficient chemical processes. The unique advantages of SBACs, compounded by their diverse applications and adaptability in various reaction conditions, position them as a valuable alternative to traditional homogeneous acid catalysts. With ongoing research, the future holds promise for even more innovative solid acid systems, contributing to sustainable industrial practices and enhancing the field of catalysis. In the last two decades, various types of SBACs, including phosphates, sulfated metal oxides, heteropoly acids, and acidic resins, have attracted the attention of many researchers due to their recyclability and outstanding activity [5]. Among the types of Brønsted acid catalysts, there are a number of them, including silica-bonded n-propyl sulfamic acid (SBNPSA) [6], silica sulfuric acid (SSA) [7], PPF-SO_3_H [8], p-Toluenesulfonic acid (p-TsOH) [9], cellulose sulfuric acid (CSA) [10] and organosilane sulfonated graphene oxide (SSi–GO) [11]. Although these catalysts have useful applications such as reusability and recyclability, short reaction time and good yield. However, they have the disadvantages of expensive reagents, multi-step synthesis, incompatibility with other functional groups and product separation with toxic organic solvents, which their use is limited due to environmental issues. Therefore, it is very important to find and discover a new support for Brønsted acid catalysts that provides the desired products with good catalytic activity and performance [[6], [7], [8], [9], [10], [11], [12]].

Multicomponent reactions (MCRs) offer an efficient and sustainable approach to synthesis, and integrating greener solvents into these processes further enhances their environmental and economic benefits [13]. The combination of MCRs and sustainable solvents promotes a shift towards greener chemistry, aligning with the goals of reducing waste, improving sustainability, and fostering safer chemical practices. As research continues to advance in this area, the development of novel MCR methodologies that embrace greener solvents [14]. MCRs are one-pot methods in which two or more atoms of beginning materials combine to form the final product. Also, MCRs have prominent features such as high efficiency, convergence, atom economy and high bond formation index (BFI) [15].

**Fig. 1:**
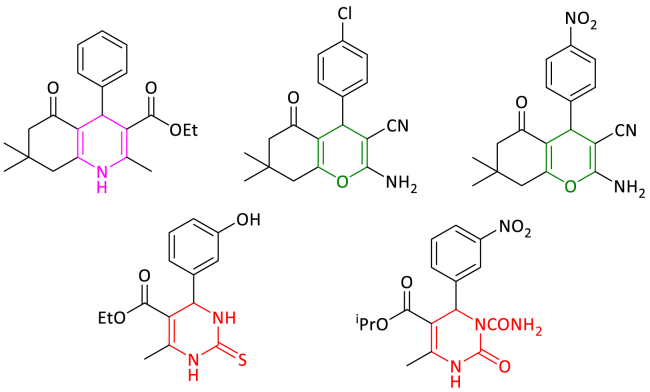
Pharmacologically active polyhydroquinolines, 2-amino-3-cyano-4H-chromenes and 3,4-dihydropyrimidine-2(1H)-one/thiones.

Heterocyclic compounds, which focus on the field of medicinal chemistry and drug discovery, form the foundational structure of a wide range of medicinal compounds and biologically active molecules, which include antiviral agents, antibiotics, and anticancer drugs [16]. A wide range of important biomedical compounds are related to polyhydroquinoline derivatives. These compounds have many uses, including vasodilator, hepatoprotective, geroprotective, antidiabetic, bronchodilator, antitumor, calcium channel blocker, analgesic and anti-inflammatory. Several techniques have been developed for the synthesis of polyhydroquinoline heterocycles, but they have disadvantages such as vigorous reaction conditions, unsafe catalysts, and tedious workup methods, so it is very important to provide green methods [17]. Another important group of heterocyclic compounds is benzopyran and its derivatives, which have attracted the attention of many researchers. Benzopyrans exist in the structure of several natural products and have attracted many medicinal and biological properties. These heterocyclic frameworks have different uses in the treatment of diseases such as Huntington, schizophrenia, Alzheimer, myoclonus, Down syndrome and Parkinson. Further, other applications of benzopyran derivatives include their use in agricultural chemicals, perfumes, food as additives, pigments, cosmetics, and photoactive substances [18]. 3,4-dihydropyrimidine-2(1H)-one/thiones are the other group of heterocyclic compounds known to undergo bigenelli reactions. These compounds and their derivatives have many applications in biological activities such as antibacterial, antimalarial, antitumor, anti-inflammatory and antiviral. Bigenelli reaction is one of the most famous multicomponent reactions, which is synthesized from the condensation between α, β-ketoesters and urea/thiourea and aldehydes. Previously, a number of solvents such as toluene, acetonitrile, tetrahydrofuran and dioxane as well as mineral acids were used for the synthesis of 3,4-dihydropyrimidine-2(1H)-one/thiones. However, these solvents are volatile, flammable, toxic and non-renewable. Furthermore, these acids have issues such as difficult isolation, strong acidic conditions, and low yields of products. Therefore, the synthesis of the mentioned heterocyclic compounds with high energy efficiency, the usage of green solvent, atomic economy and low-cost method which are some of the criteria of green chemistry is investigated under the conditions of room temperature and water. Fig. 1 indicates some pharmacologically active polyhydroquinolines, 2-amino-3-cyano-4H-chromenes and 3,4-dihydropyrimidine-2(1H)-one/thiones derivative [12,19,20].

Carbon materials, including carbon nanotubes [21], zeolites [22], graphene [21], and carbon nitride [21], graphitic carbon nitride (g-C_3_N_4_) [23] have garnered significant attention in various fields, particularly in heterogeneous catalysis. Their unique properties, such as high surface area, electrical conductivity, mechanical strength, and chemical stability, make them suitable candidates for enhancing catalytic processes.

Mitigating the environmental impact of industrial chemical processes is a critical goal in modern chemistry, particularly in the context of green chemistry and catalysis. Catalytic reactions are generally preferred over stoichiometric reactions in green chemistry, as they can improve efficiency and reduce waste. The development of truly eco-friendly catalytic processes remains an ongoing challenge. Heterogeneous catalysis is particularly advantageous, as it facilitates catalyst recovery and reduces waste generation compared to homogeneous catalysts. The overall aim is to design catalytic processes that have a much lower environmental impact than traditional stoichiometric chemical methods. This captures the essence of the environmental considerations and strategies in modern catalytic chemistry. Continued research and innovation in this area is crucial for developing more sustainable industrial chemical processes [24].

Natural asphalt (NA), which consists of polycyclic aromatic structures and aliphatic moieties, contains 70–80 % carbon, 15 % hydrogen along with traces of oxygen, sulfur and nitrogen. The three largest sources of NA in the world are in the United States, Canada, and Iran respectively. Although due to its natural and non-toxic nature, this source of asphalt has valuable applications in various fields such as coke production, asphalt, dyes, drilling mud and foundry industries. However, it has been emerging for a decade in laboratory research on its nature in organic reactions and other laboratory work. Also, due to its useful features such as availability, low cost, excellent stability, specific surface area, good thermal stability and high carbon content, NA can be used as a suitable candidate carbon support for the preparation of various catalysts [[25], [26], [27]].

In the continuity of our study on the nature of NA and its further applications in research and organic reactions, for the first time, we succeeded in nitrating the NA support and then converting the NA-NO_2_ into sulfamic acid (NA-NHSO_3_H). Finally, it is used as a Brønsted acid catalyst in a number of organic reactions including polyhydroquinoline, tetrahydrobenzo[b]pyran and 3,4-dihydropyrimidine-2(1H)-one/thione in water and room temperature in line with the goals of green chemistry. Among the advantages of acidic Brønsted catalyst, we mention the excellent yield of products, less acidic waste, recyclability, solid, simple separation, high catalytic activity and production of heterocyclic compounds on a gram-scale (20 mmol).

## 2 Experimental

### 2.1 Material and apparatus

All chemicals and solvents were purchased from Sigma-Aldrich and Merck chemical companies. Natural asphalt was bought from natural bitumen mines in the west of Iran. NA was utilized as a powdery, black material with a melting point above 180 °C and an ash content of 6 %. Thin layer chromatography (TLC) was monitored for reactions on Polygram SILG/UV254 silica-gel plates. FT-IR spectra on KBr pellets were performed by FT-IR, VERTEX 70, Bruker, Germany, spectroscopy. Scanning electron microscope (SEM) images were recorded on FE-SEM, TESCAN MIRA III, Czech. Energy-dispersive X-ray -Map (EDX- Map) spectroscopy was utilized using the Czech TESCAN instrument. The nitrogen adsorption-desorption isotherm was conducted at 77 K by BET, Micromeritics, Asap2020, USA. The instruments’ thermogravimetric analysis (TGA) from 25 °C to 800 °C was measured by TGA, NETZSCH, Germany and TEM of the NA-NHSO_3_H was carried out with a Philips-EM 208S TEM. ^1^H NMR and ^13^C NMR spectra of synthesized products in the DMSO-d_6_ solutions were determined using a Bruker DRX-250 AVANCE instrument through TMS as an internal standard.

### 2.2 Nitration of NA; Synthesis of NA-NO_2_

A mixture of 17.5/20 mL of nitric acid/sulfuric acid in a 250 mL flask was stood at 0 °C for 15 min, then 2 g of NA was slowly added to it. After 30 min, the reaction temperature was increased to 60 °C. The reaction continued for 5 h. Finally, 300 mL of distilled water was added to the reaction mixture. After filtering and washing with water (3 × 15), it was dried in the oven at 80 °C for 3 h [28].

### 2.3 Reduction of NA-NO_2_; Synthesis of NA-NH_2_

**Fig. 2:**
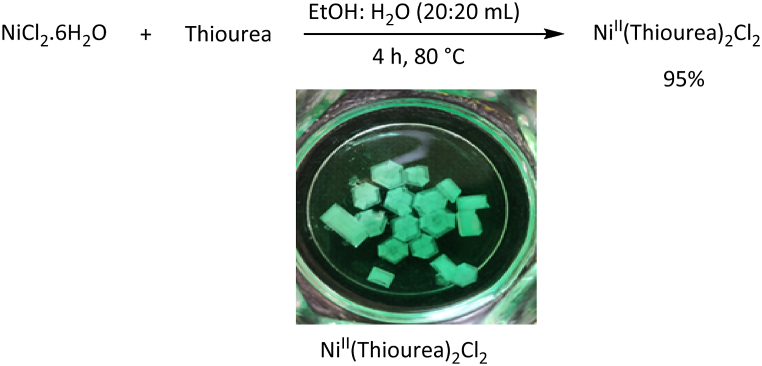
Reaction of NiCl_2_·6H_2_O with thiourea.

Complex production: In a 50 mL flask equipped with a magnetic stirrer, thiourea (0.02 mol, 1.52 g) and EtOH: H_2_O (20:20 mL) were added. After dissolving thiourea at 50 °C, 0.005 mol (1.18 g) of NiCl_2_·6H_2_O was poured into it. The reaction mixture was stirred for 4 h at 80 °C. After completing the reaction, the mixture was stand at room temperature for one week to evaporate ethanol, and the nickel complex was obtained. Finally, EtOH was used several times to wash it. The pure complex was prepared with a yield of 95 % (Fig. 2) [29].

To convert the -NO_2_ group into -NH_2_, 1.0 g of NA-NO_2_ and 1 g of nickel complex were poured into a 100 mL two-neck round-bottom flask. The reaction mixture was stirred for 20 min. To control the H_2_ gas, the reaction temperature was lowered to 0 °C, and then NaBH_4_ (0.7 g) was added to it. After 2 h, the reaction mixture was stood at room temperature for 24 h to dry.

### 2.4 Conversion of NA-NH_2_ to NA-NHSO_3_H; Synthesis of NA-NHSO_3_H

Two methods were applied for the synthesis of NA-NHSO_3_H:

In the first method, SO_3_ was produced first. After preparing SO_3_, 0.20 g of NA-NH_2_ was added to 2 mL of SO_3_ and the reaction mixture was stirred for 4 h at room temperature.

**Scheme 1:**
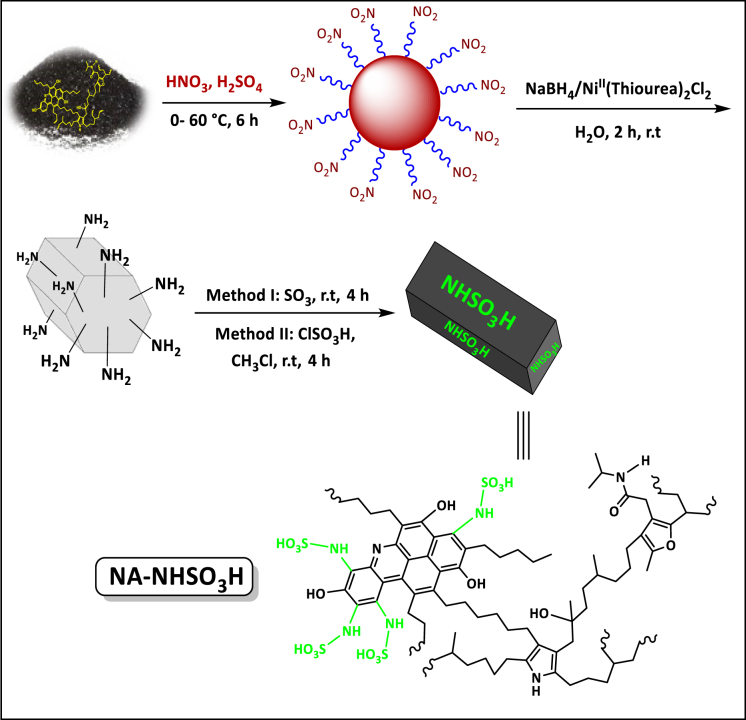
Synthesis of NA-NHSO_3_H.

In the second method, 1 g of NA-NH_2_ was added to 15 mL of chloroform. Then 0.5 mL chlorosulfonic acid was added dropwise for 20 min. Afterward, the reaction mixture was stirred for 4 h at ambient temperature. After the mentioned time, the reaction mixture was washed with water and dried at room temperature (Scheme 1).

**Fig. 3:**
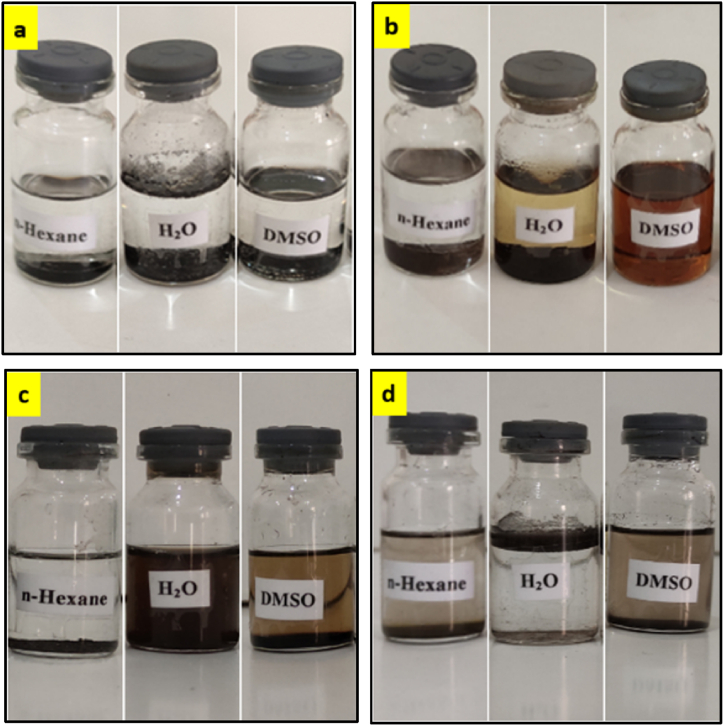
Comparison of solubility between NA (a), NA-NO_2_ (b), NA-NH_2_ (c) and NA-NHSO_3_H (d).

The solubility of four samples NA, NA-NO_2_, NA-NH_2_, and NA-NHSO_3_H has presented in Fig. 3a-d. NA (Fig. 3a) and NA-NHSO_3_H (Fig. 3d) are insoluble in protic (H_2_O), aprotic (DMSO) and nonpolar (n-hexane) solvents. However, NA-NO_2_ is fully soluble in dimethyl sulfoxide (DMSO) but not in H_2_O and n-hexane (Fig. 3b). Conversely, the NA-NH_2_ sample is generally insoluble in non-polar or aprotic solvents but soluble in H_2_O (Fig. 3c).

### 2.5 General procedure for the preparation of polyhydroquinoline

A mixture of aldehyde (1 mmol), ethyl acetoacetate (1 mmol, 130 mg), dimedone (1 mmol, 140 mg), NH_4_OAC (1.2 mmol, 93 mg) and NA-NHSO_3_H (10 mg) was stirred in 1 mL H_2_O for an appropriate time. TLC was applied to monitor the progress of the reaction. After consuming the starting material, the desired catalyst was separated from the product by filtration and finally, the pure product was obtained in hot EtOH through recrystallization.

### 2.6 General procedure for the preparation of tetrahydrobenzo[b]pyran

A mixture of aromatic aldehydes (1 mmol), dimedone (1 mmol, 140 mg), malononitrile (1 mmol, 66 mg) and NA-NHSO_3_H (10 mg) was stirred in 1 mL H_2_O for the required time. The progress of reaction was monitored by TLC. After the completion of the reaction, the catalyst was filtered and separated from the reaction product. Eventually, pure tetrahydrobenzo[b]pyran products were obtained by recrystallization in hot EtOH.

### 2.7 General procedure for the preparation of 3,4-dihydropyrimidine-2(1H)-one/thione

A molar ratio mixture of aldehyde (1 mmol), urea/thiourea (1.2 mmol, 72/91 mg) and ethyl acetoacetate (1 mmol, 130 mg) in the presence of NA-NHSO_3_H (10 mg) as catalyst was added in H_2_O (1 mL) at room temperature. The completion of the reaction was assayed by TLC. After that, the catalyst was separated by filtration from the product. Finally, pure products were prepared by recrystallization in hot EtOH.

## 3 Results and discussion

### 3.1 Catalyst characterization

**Fig. 4:**
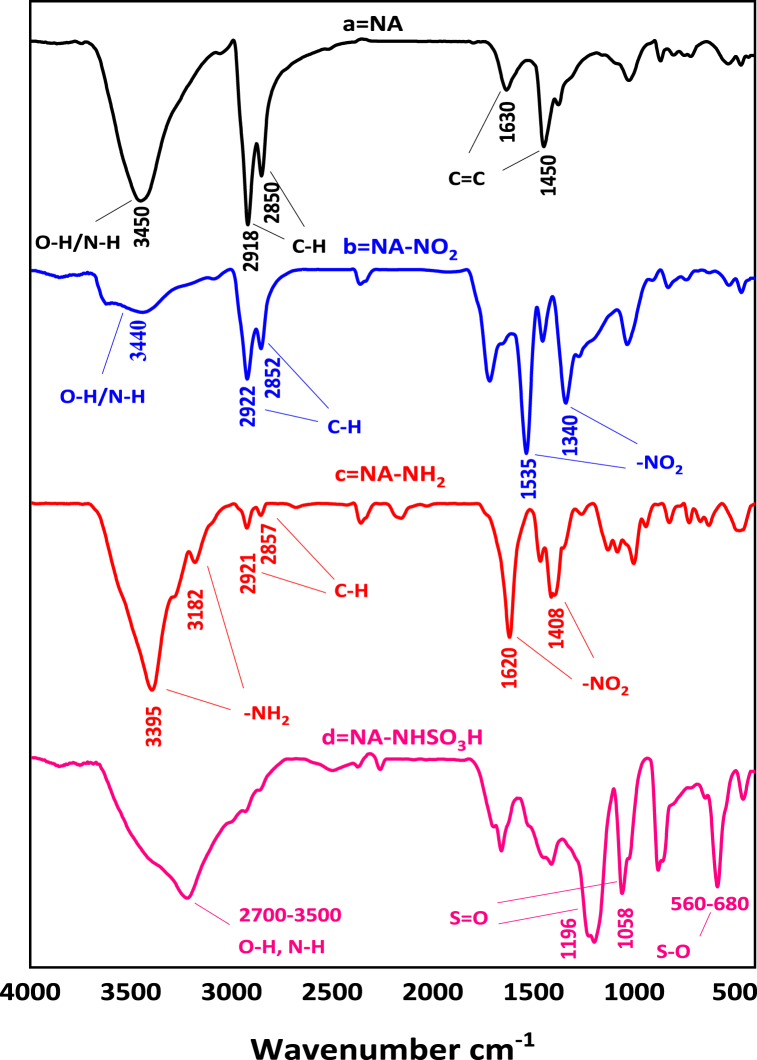
FT-IR spectra of NA (a), NA-NO_2_ (b), NA-NH_2_ (c) NA-NHSO_3_H (d).

After the successful synthesis of NA-NHSO_3_H, various techniques including FT-IR, SEM, EDX-Map, BET, TGA and TEM were used to characterize it. The FT-IR spectra of NA (a), NA-NO_2_ (b), NA-NH_2_ (c) and NA-NHSO_3_H (d) are revealed in Fig. 4. Accordingly, the spectra (a-d) show 3331-3450 cm^−1^ peaks related to O-H and N-H groups. Also, in all the spectra, stretching vibrations related to the aliphatic C−H group appeared in the region of 2850–2922 cm^−1^, but in the d spectrum, it was covered by acidic groups. Symmetrical and asymmetric stretching vibrations at 1340–1535 cm^−1^ in Fig. 4b exhibit the presence of the -NO_2_ group in the NA-NO_2_. In Fig. 4c, the characteristic absorption peaks of amine with the reduction of the nitro group were indicated at 3182 and 3395 cm^−1^. Finally, the appearance of a new absorption band at the range of 1058–1196 cm^−1^ into the S=O and a strong stretching band at 560-680 cm^−1^ is attributed to S-O, which confirms the presence of the sulfonic acid functional group (Fig. 4d).

**Fig. 5:**
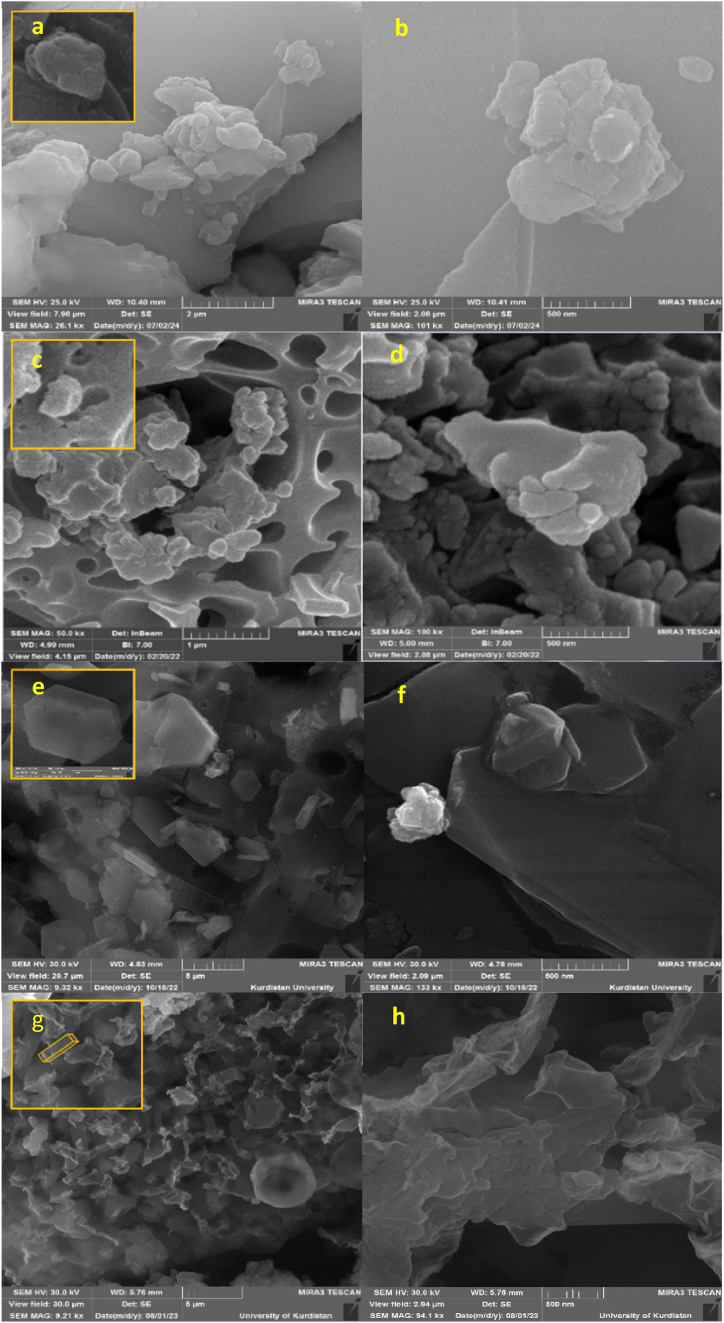
SEM images of NA (a, b), NA-NO_2_ (c, d), NA-NH_2_ (e, f) NA-NHSO_3_H (g, h).

**Fig. 6:**
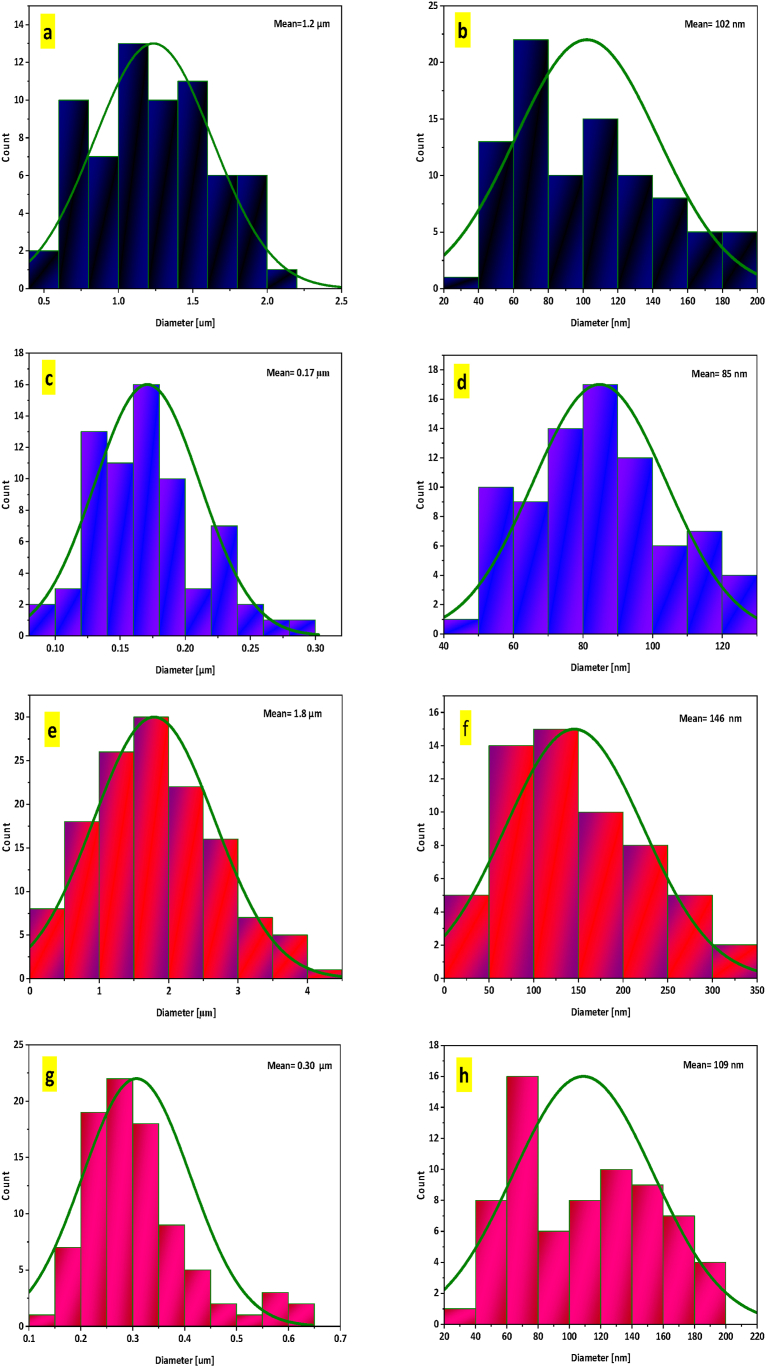
Histograms of NA (a, b), NA-NO_2_ (c, d), NA-NH_2_ (e, f) NA-NHSO_3_H (g, h).

Morphology, particle size and structural features of NA, NA-NO_2_, NA-NH_2_, and NA-NHSO_3_H were determined by SEM analysis at the micro-nano scale in Fig. 5(a–h) and S1-S4. Also, their histogram and particle size are exhibited in Fig. 6a-h. It is clearly known that NA (Fig. 5a and b) and NA-NO_2_ (Fig. 5c and d) have spherical structures and their particle size average is 102 and 85 nm, respectively. In NA-NH_2_ (Fig. 5e and f), which has hexagonal particles, the particle size average is 146 nm. Their histograms of NA, NA-NO_2_ and NA-NH_2_ are shown in Fig. 6a-b, 6(c-d) and 6(e-f), respectively. But in NA-NHSO_3_H (Fig. 5g and h), which is what we are considering, when -SO_3_H groups are stabilized on the surface of NA-NH_2_, the structure and size of the particles change and the size average of the particles reaches from 146 nm to 109 nm (Fig. 6g and h). It is also beautifully evident that NA-NHSO_3_H is formed from cubic crystals. Additionally, it can be mentioned that the distribution of uniform particles is obvious in them.

**Fig. 7:**
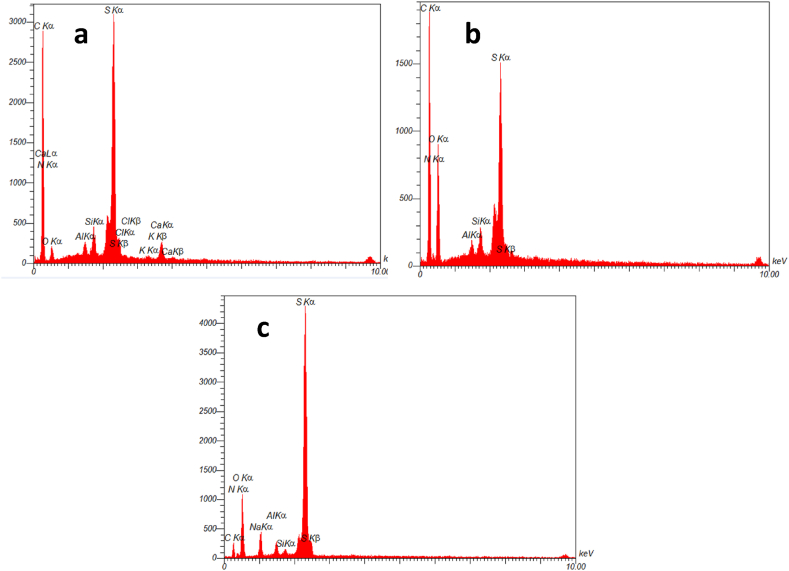
EDX analysis of NA (a), NA-NO_2_ (b) and NA-NHSO_3_H (c).

**Fig. 8:**
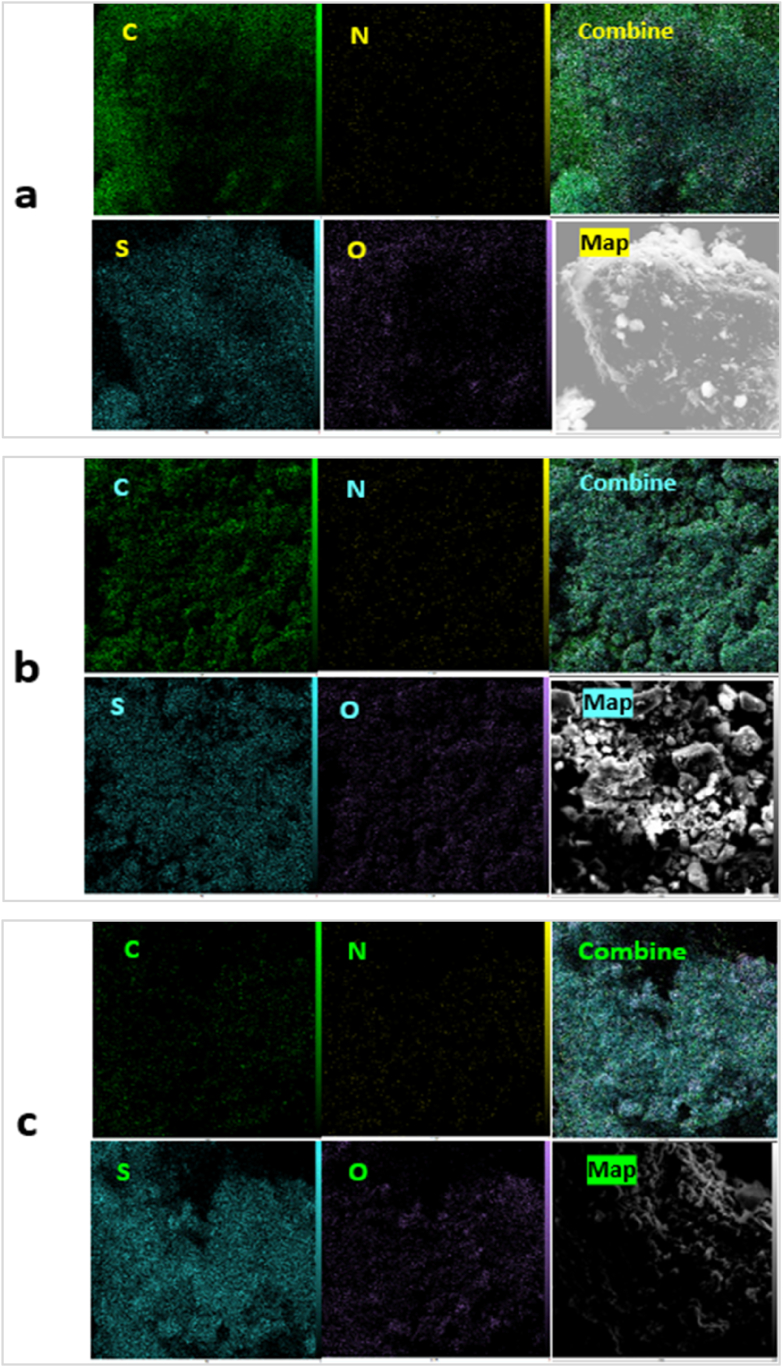
Elements mapping analysis of NA (a), NA-NO_2_ (b) and NA-NHSO_3_H (c).

To investigate the elemental characterization of NA, NA-NO_2_ NA-NHSO_3_H, EDX and elemental mapping analysis was utilized (Fig. 7, Fig. 8). As shown by the EDX result, a good elemental distribution was obtained in NA (Fig. 7a), NA-NO_2_ (Fig. 7b) as well as NA-NHSO_3_H catalyst (Fig. 7c). Also, the map images of the elements (C, S, N and O) in NA (Fig. 8a), NA-NO_2_ (Fig. 8b) and NA-NHSO_3_H (Fig. 8c) have suitable dispersion and homogeneity. In this sense, the uniform distribution of -SO_3_H sites on the NA-NH_2_ surface has a significant effect on the catalyst performance due to the good availability of catalytic sites. It can be concluded that the results of elemental mapping analysis are consistent with their EDX. The amount of elements in NA, NA-NO_2_ and NA-NHSO_3_H samples using EDX and their relationship with elemental mapping are shown in Table 1.

**Table 1:** Elemental analysis of NA, NA-NO_2_ and NA-NHSO_3_H by using EDX analysis.

**Compound**	**Element**	**K**	**K**_**r**_	**W [%]**	**A [%]**
NA	C	0.3640	0.5052	92.75	95.95
	N	0.0000	0.0000	0.00	0.00
	O	0.0056	0.0030	4.17	3.23
	S	0.0516	0.0277	3.07	1.19
	Total	1.0000	0.5359	100	100
NA-NO_2_	C	0.8713	0.2948	60.17	66.33
	N	0.0214	0.0072	10.58	10.01
	O	0.0751	0.0254	27.95	23.13
	S	0.0323	0.0109	1.29	0.53
	Total	1.0000	0.03383	100	100
NA-NHSO_3_H	C	0.3640	0.0761	33.30	40.72
	N	0.0649	0.0136	13.20	13.84
	O	0.2587	0.0541	45.52	41.79
	S	0.3124	0.0653	7.98	3.66
	Total	1.0000	0.2090	100	100

**Fig. 9:**
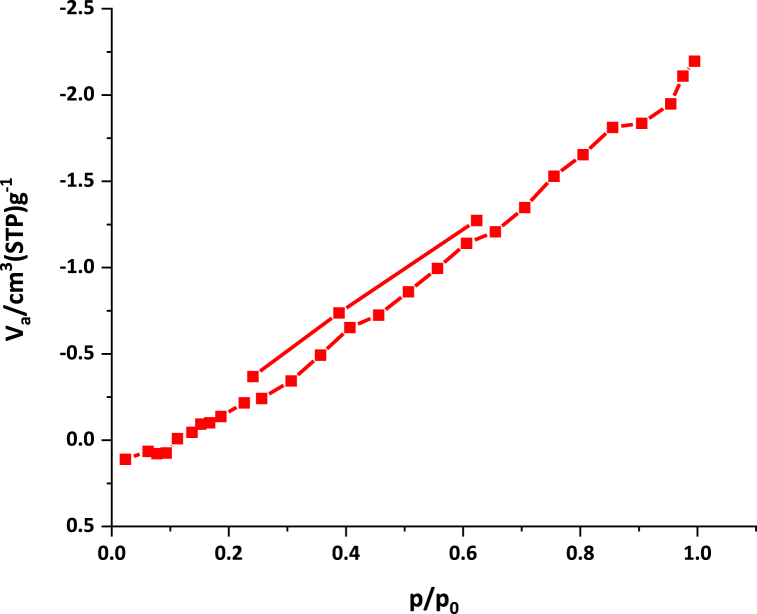
The N_2_ adsorption–desorption isotherm for NA-NHSO_3_H.

Fig. 9 shown the nitrogen adsorption–desorption isotherms of NA-NHSO_3_H. Based on the data obtained from BET, the catalyst showed type III isotherm. The data related to N_2_ adsorption-desorption isotherm of NA-NHSO_3_H catalyst are shown in Table 1. The surface area and mean pore diameter for NA are 10.49 m^2^ g^1^ and 10.65 nm, respectively. But for NA-NHSO_3_H, these values decreased to 1.33 m^2^ g^1^ and 1.22 nm. This means that the -SO_3_H groups on the surface of the NA wall were covalently bonded (Table 2).

**Table 2:** Textural properties of NA-NHSO_3_H obtained by nitrogen adsorption-desorption analysis.

**Sample name**	**S**_**BET**_**(m**^**2**^**.g**^**1**^**)**	**D**_**BJH**_**(nm)**
NA	10.49	10.65
NA-NHSO_3_H	1.33	1.22

**Fig. 10:**
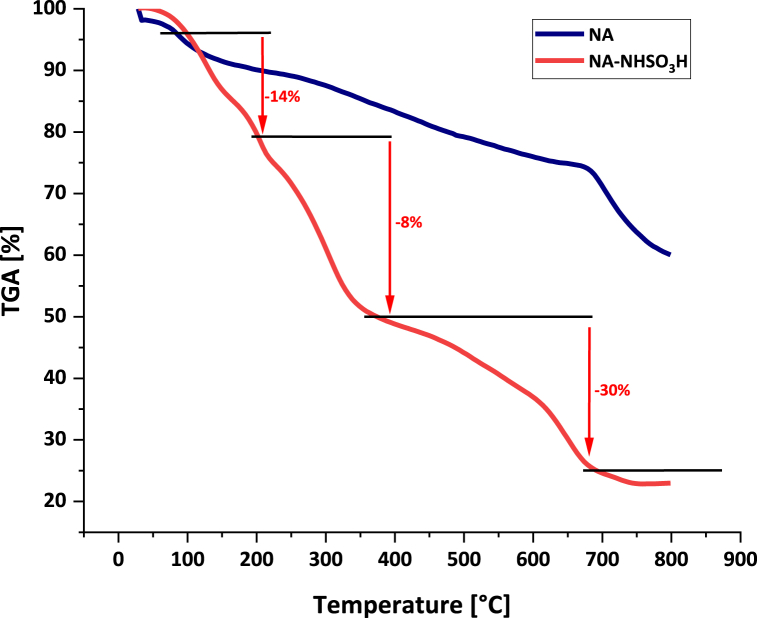
Thermogravimetric curves of NA and NA-NHSO_3_H.

To evaluate the thermal stability of NA-NHSO_3_H and comparison with NA, the TGA analysis over the temperature range of 25–800 °C was applied (Fig. 10). The TGA curve shows the three-weight loss for NA-NHSO_3_H. The first weight loss of about 14 % occurred below 200 °C, which could be due to the removal of water and organic solvents adsorbed on the catalyst surface. The next degradation of 8 % in 200–400 is the result of the decomposition of organic groups and fragments. The final weight loss of about 30 % occurred above 400–700 °C, which is attributed to -SO_3_H groups. A comparison of NA-NHSO_3_H and NA curves showed that -SO_3_H groups are successfully stabilized on NA support through chemical bonding.

**Fig. 11:**
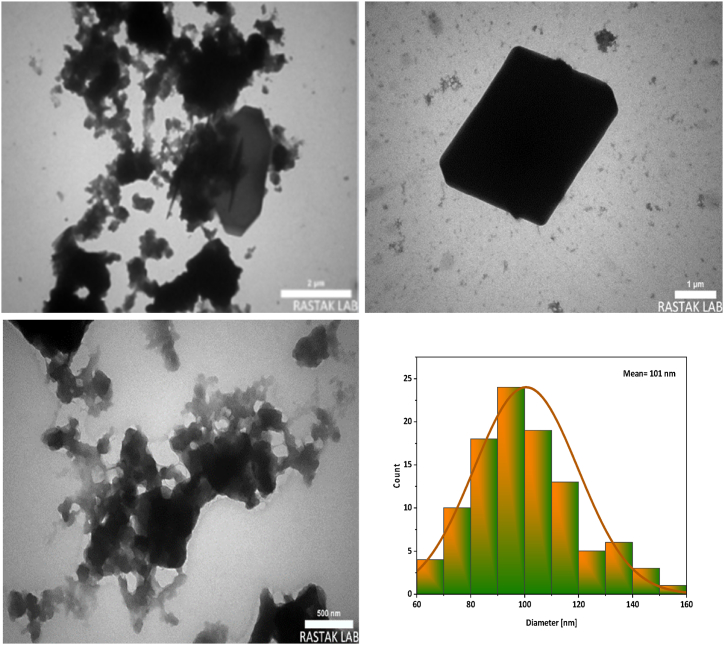
TEM images and histogram of NA-NHSO_3_H.

The size distribution and structure of NA-NHSO_3_H were analysed by TEM (Fig. 11 and Fig. S5). It is quite clear that the images obtained confirm the formation of cubic crystals in the catalyst. In addition, the histogram of particle size distribution from TEM images indicates that their average size is 101 nm. Also, the crystals were connected in a chain network, which increased the catalytic activity. The results confirmed that the size average and shape of crystals in TEM and SEM analysis are consistent.

### 3.2 Effect of pH

**Fig. 12:**
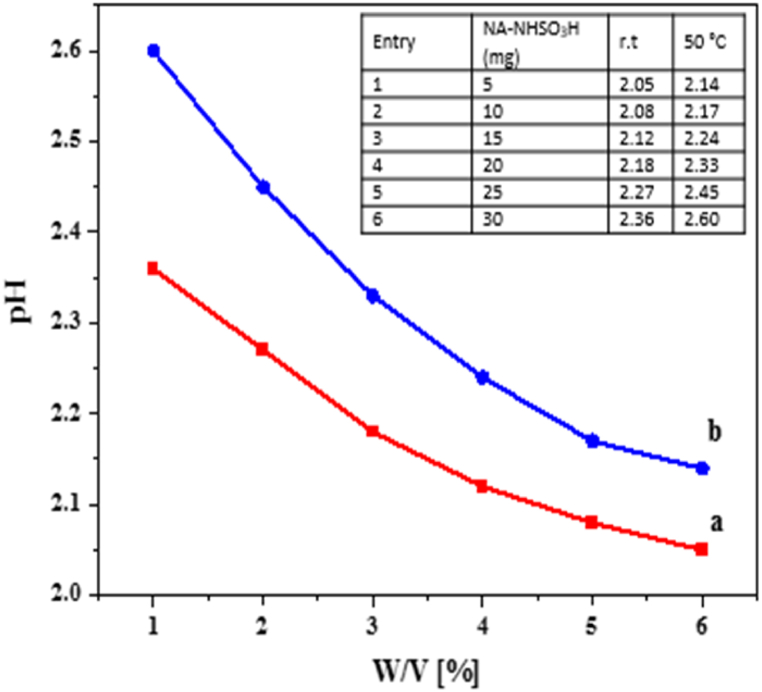
Measurement of pH of NA-NHSO_3_H catalyst at room temperature (a) and 50 °C (b).

For this purpose, two different temperatures (r.t, 50 °C) were measured to check the acidity of the NA-NHSO_3_H catalyst using a glass electrode. First 5 mg of catalyst was dispersed in 5 mL of deionized water at room temperature. And the electrode showed a value of pH = 2.05. The pH measurement continued for 5 more steps. In addition, these steps were also repeated to measure pH at a temperature of 50 °C (Fig. 12). The results showed that temperature was an influencing factor on pH value. Also, the effect of pH was evaluated using classical methods (acid-base titration).

By comparing Fig. 12 methods, it can be seen that the pH value and the acidity of the catalyst are close to each other and no significant difference was observed.

### 3.3 Catalytic studies

After the successful synthesis, evaluation and identification of NA-NHSO_3_H catalyst, its efficacy for the preparation of polyhydroquinolines, tetrahydrobenzo[b]pyrans and 3,4-dihydropyrimidine-2(1H)-one/thiones was investigated.

**Scheme 2:**
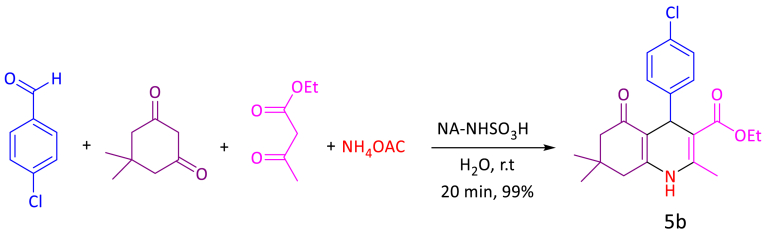
Optimal reaction condition for the synthesis of 5b.

To optimize the reaction conditions in polyhydroquinolines, different amounts of catalyst, various solvents and temperatures were evaluated (Scheme 2). First, different amounts of catalyst for the reaction of 4-chlorobenzaldehyde (1 mmol, 140 mg), dimedone (1 mmol, 140 mg), ethyl acetoacetate (1 mmol, 130 mg), ammonium acetate (1.2 mmol, 93 mg) and NA-NHSO_3_H as catalyst were checked, which 10 mg of catalyst was found to be the best amount catalyst (Scheme 2). No significant increase in product yield was observed when the amount of catalyst increased from 10 mg to 15 mg. Next, various solvents such as EtOH, PEG, solvent-free, mixture of H_2_O: EtOH (2:1 mL) and H_2_O were checked. We also investigated various temperatures for the model reactions; the results suggest that r. t is a better temperature for the reactions. The result clearly exhibits that 10 mg of NA-NHSO_3_H in H_2_O at room temperature is the ideal condition to produce an excellent yield of the desired product (Table 3, entry 3, 5b).

**Table 3:** Optimization of reaction conditions for synthesis of **5b**, **7b**, **9b**; catalyzed by NA-NHSO_3_H.

Entry	NA-NHSO_3_H (mg)	Solvent	Temp (°C)	Yield^a^ (%)		
				5b^b^	7b^c^	9b^d^
1	0	H_2_O	r.t	15	43	Trace
2	8	H_2_O	r.t	89	95	96
3	10	H_2_O	r.t	99	99	98
4	15	H_2_O	r.t	95	93	90
5	10	Solvent-free	r.t	80	88	87
6	10	H_2_O: EtOH^e^	r.t	93	94	88
7	10	H_2_O	50	88	91	89
8	10	EtOH	r.t	91	89	84
9	10	PEG	r.t	80	86	85
10	10	H_2_O^f^	r.t	24	27	12

Note: The bold values represent optimized reaction conditions.

Reaction conditions.

a Isolated yield.

b 4-chlorobenzaldehyde (1 mmol, 140 mg), dimedone (1 mmol, 140 mg), ethyl acetoacetate (1 mmol, 130 mg), ammonium acetate (1.2 mmol, 93 mg), 20 min.

c 4-chlorobenzaldehyde (1 mmol, 140 mg), dimedone (1 mmol, 140 mg), malononitrile (1 mmol, 66 mg), 15 min.

d 4-chlorobenzaldehyde (1 mmol, 140 mg), ethyl acetoacetate (1 mmol, 130 mg), urea/thiourea (1.2 mmol, 72/91 mg), 20 min; catalyst (mg) and solvent (1 mL).

e Ratio: 2:1 mL.

f The reaction catalyzed by NA-NH_2_.

**Scheme 3:**
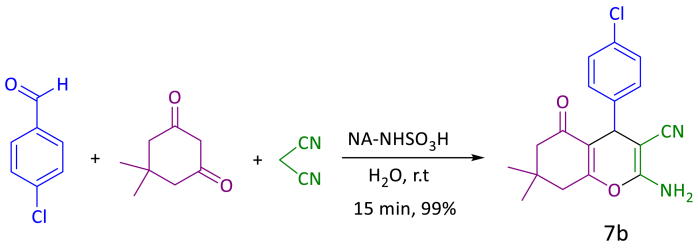
Optimal reaction condition for the synthesis of 7b.

In the next part, we also optimized various effective factors such as the amount of catalyst, type of solvent and temperature for the synthesis of tetrahydrobenzo[b]pyrans. For this purpose, the reaction of 4-chlorobenzaldehyde (1 mmol, 140 mg), dimedone (1 mmol, 140 mg) and malononitrile (1 mmol, 66 mg) in the presence of NA-NHSO_3_H catalyst was chosen as the model reaction (Scheme 3). With the evaluation of the amount of catalyst, solvents and the reaction temperature, we found that 10 mg of catalyst, H_2_O and r. t were the most effective condition for the synthesis tetrahydrobenzo[b]pyrans (Table 3, entry 3, 7b).

**Scheme 4:**
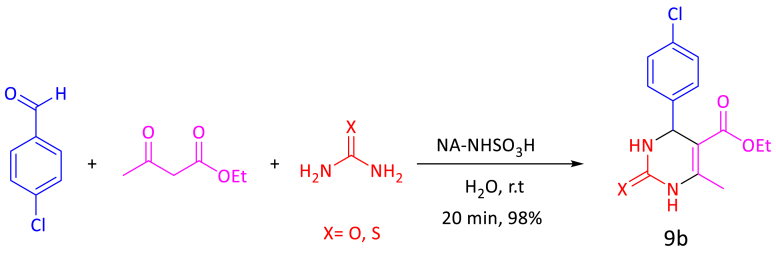
Optimal reaction condition for the synthesis of 9b.

In the last part, we optimized the above effective agents for the synthesis of 3,4-dihydropyrimidine-2(1H)-one/thiones. To understand the efficiency of the catalyst in the synthesis of 3,4-dihydropyrimidine-2(1H)-one/thiones, the condensation of 4-chlorobenzaldehyde (1 mmol, 140 mg), ethyl acetoacetate (1 mmol, 130 mg), urea/thiourea (1.2 mmol, 72/91 mg) was determined in the presence of NA-NHSO_3_H in H_2_O at room temperature as a model reaction (Scheme 4). By changing the effective factors (amount of catalyst, solvent and temperature), we found that the best conditions for the synthesis of and 3,4-dihydropyrimidine-2(1H)-one/thiones were in the presence of 10 mg of NA-NHSO_3_H and H_2_O at room temperature (Table 3, entry 3, 9b).

Moreover, to investigate the effect of the -SO_3_H group in the synthesized catalyst in promoting the reactions, the reaction was investigated in the presence of NA-NH_2_. That result showed that the effect and acid property of this group is very important in the catalytic activity (Table 3, entry 10).

After optimizing the reaction conditions of polyhydroquinoline, tetrahydrobenzo[b]pyran and 3,4-dihydropyrimidine-2(1H)-one/thione, a range of electron-withdrawing and electron-donating aldehydes were studied and derivatives of 5(a-k), 7(a-l) and 9(a-p) were obtained in excellent yield as summarized in Table 4, Table 5, Table 6. From Table 4, Table 5, Table 6, it is obvious that various derivatives of polyhydroquinolines, tetrahydrobenzo[b]pyrans and 3,4-dihydropyrimidine-2(1H)-one/thiones were synthesized with reaction time, yield and melting point values.

**Table 4:**
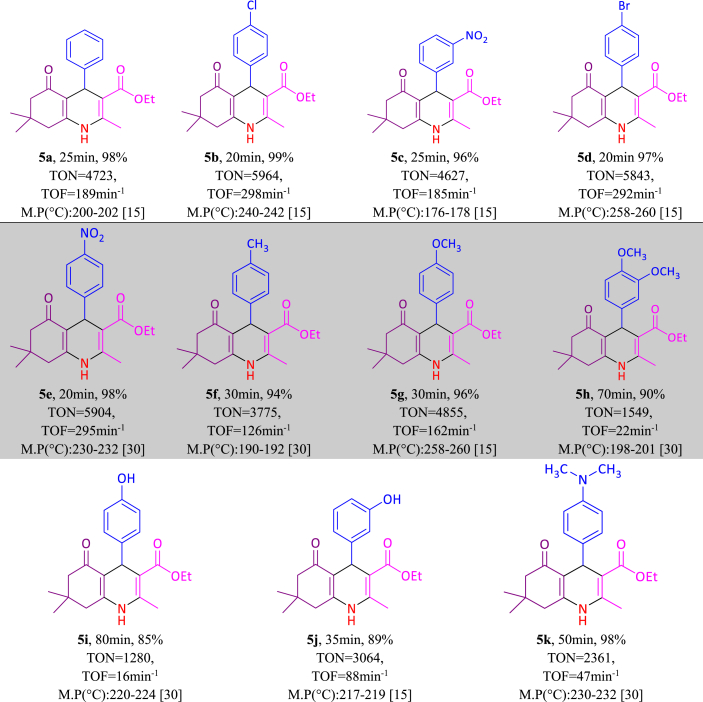
One-pot synthesis of **5(a**–**k)** derivatives in the presence of NA-NHSO_3_H^a^.

a Reaction conditions: aromatic aldehyde (1 mmol), dimedone (1 mmol, 140 mg), ethyl acetoacetate (1 mmol, 130 mg), ammonium acetate (1.2 mmol, 93 mg), NA-NHSO_3_H (10 mg) at room temperature in 1 mL of H_2_O.

**Table 5:**
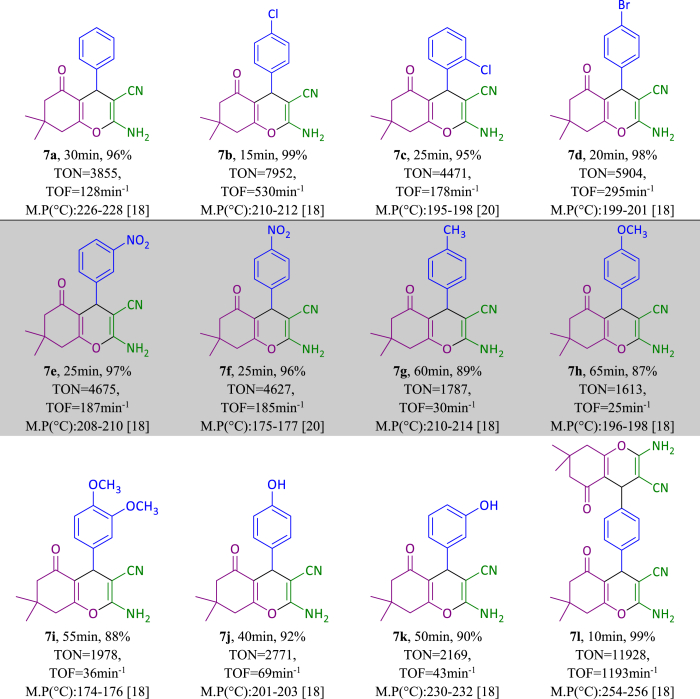
Synthesis of **9(a-p**) derivatives in the presence NA-NHSO_3_H^a^.

a Reaction conditions: aromatic aldehyde (1 mmol), dimedone (1 mmol, 140 mg), malononitrile (1 mmol, 66 mg), NA-NHSO_3_H (10 mg) at room temperature in 1 mL of H_2_O.

**Table 6:**
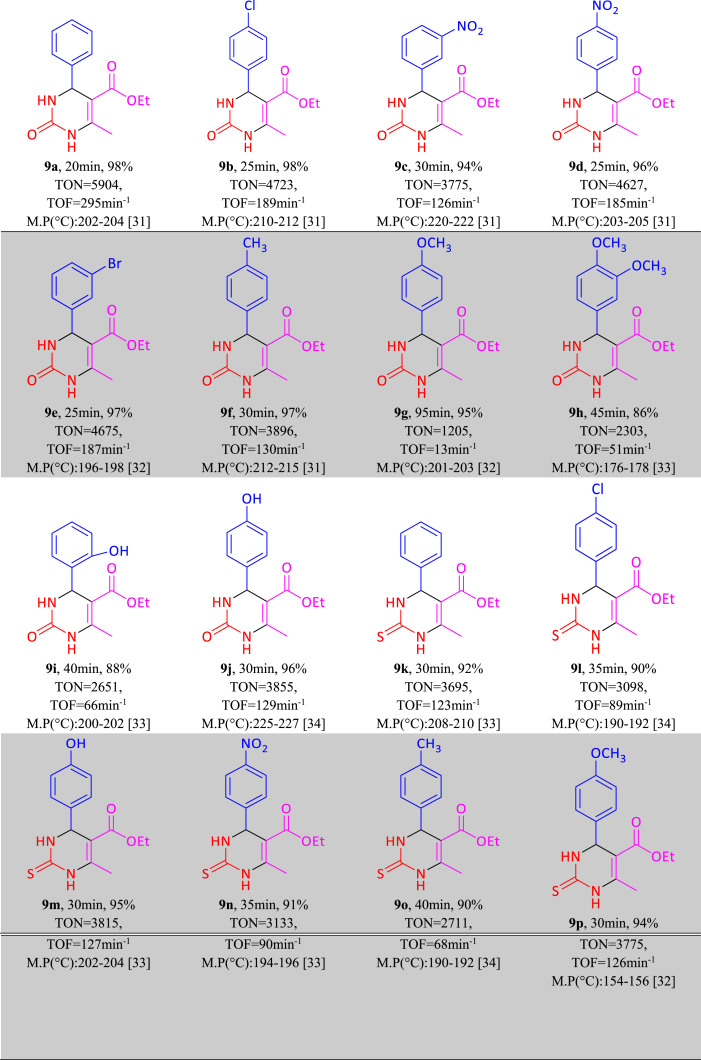
Synthesis of **9(a**–**p)** derivatives in the presence NA-NHSO_3_H^a^.

a Reaction conditions: aromatic aldehyde (1 mmol), ethyl acetoacetate (1 mmol, 130 mg), urea/thiourea (1.2 mmol, 72/91 mg), NA-NHSO_3_H (10 mg) at room temperature in 1 mL of H_2_O.[[30], [31], [32], [33], [34]]

### 3.4 Reaction mechanism

**Scheme 5:**
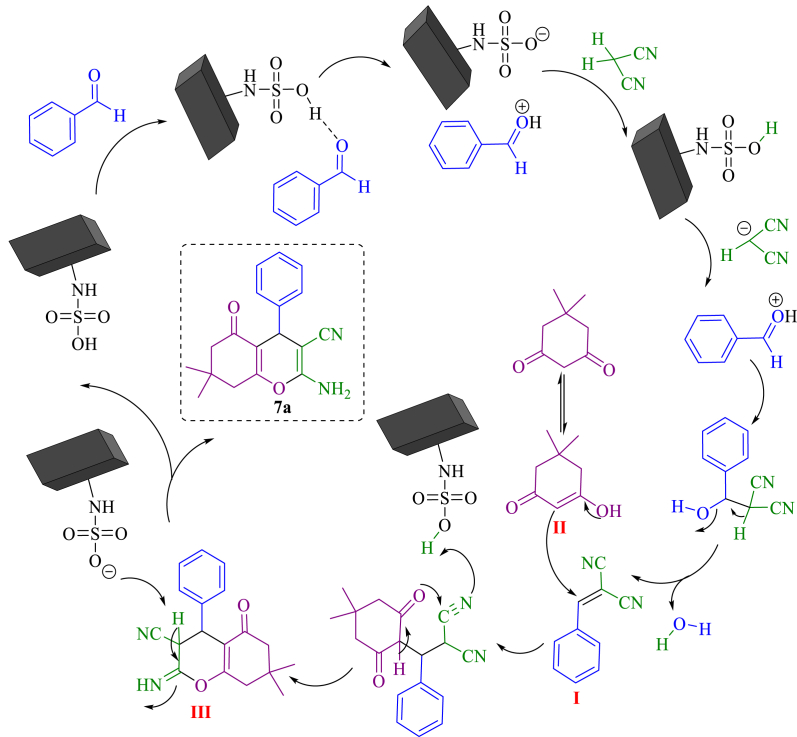
The proposed mechanism for the synthesis of 7a in the presence of NA-NHSO_3_H.

Reactions mechanism of polyhydroquinoline, tetrahydrobenzo[b]pyran and 3,4-dihydropyrimidine-2(1H)-one/thione from a general sequence of knoevenagel condensation−michael Addition−cyclization Reactions follow. Based on previous investigations, the possible mechanism for the synthesis of tetrahydrobenzo[b]pyrans in the presence of NA-NHSO_3_H has been explained (Scheme 5). First, the aldehyde is activated by the catalyst through hydrogen bonding. Then nucleophilic addition of malononitrile produces intermediate Knoevenagel condensation I (arylidenemalononitrile). In the next step, the enolization of dimedone with intermediate II leads to the preparation of intermediate III. Finally, tetrahydrobenzo[b]pyran is formed by intramolecular cyclization.

The catalytic activity of the NA-NHSO_3_H catalyst was assessed in the polyhydroquinolines, tetrahydrobenzo[b]pyrans and 3,4-dihydropyrimidine-2(1H)-one/thiones reactions when scaled up to 20 mmol. The results illustrated that after 60, 50 and 90 min, 95, 97 and 94 % yields were obtained for reactions 5b, 7b and 9c, respectively. These gratifying results illustrate the possibility of its industrial production with the excellent activity of the NA-NHSO_3_H catalyst in the desired reactions on a gram-scale.

The products were identified by FT-IR, ^1^H NMR, and ^13^C NMR (Figs. S6–S10).

### 3.5 Comparison of efficiency of catalyst

In studies related to catalysts, two parameters Turnover Number (TON) and Turnover Frequency (TOF) are mentioned. The TON represents the total number of substrate molecules that a catalyst can convert to product per molecule of catalyst, typically represented by the yield (i.e., the amount of product formed) divided by the amount of catalyst used in moles. This is as given in equation (1).

(1) $$TON=\frac{Yield}{Amount\;of\;Catalyst\;\left(mol\right)\;}$$

The TOF represents the number of substrate molecules that a catalyst can convert to product per molecule of catalyst per unit time. This is calculated by dividing the Turnover Number (TON) by the time taken for the reaction (equation (2)). A higher TOF value indicates a more efficient catalyst, as it means that the catalyst can facilitate a larger number of reactions per site per unit time.

(2) $$TOF=\frac{TON}{Time}$$

TON and TON values related to polyhydroquinoline, tetrahydrobenzo[b]pyran and 3,4-dihydropyrimidine-2(1H)-one/thione derivatives are shown in Table 1, Table 2, Table 3

### 3.6 Recyclability of catalyst

**Fig. 13:**
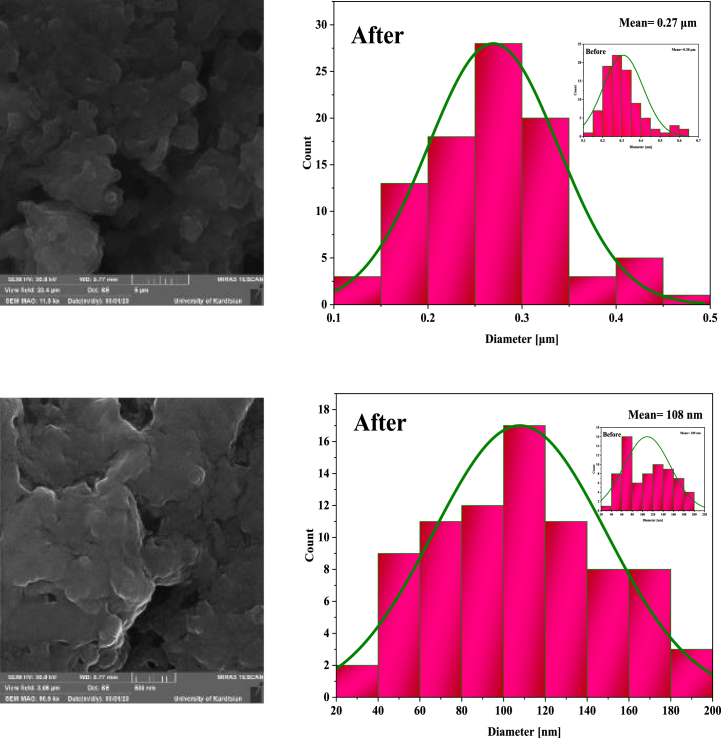
SEM images and histograms of NA-NHSO_3_H after recovery.

**Fig. 14:**
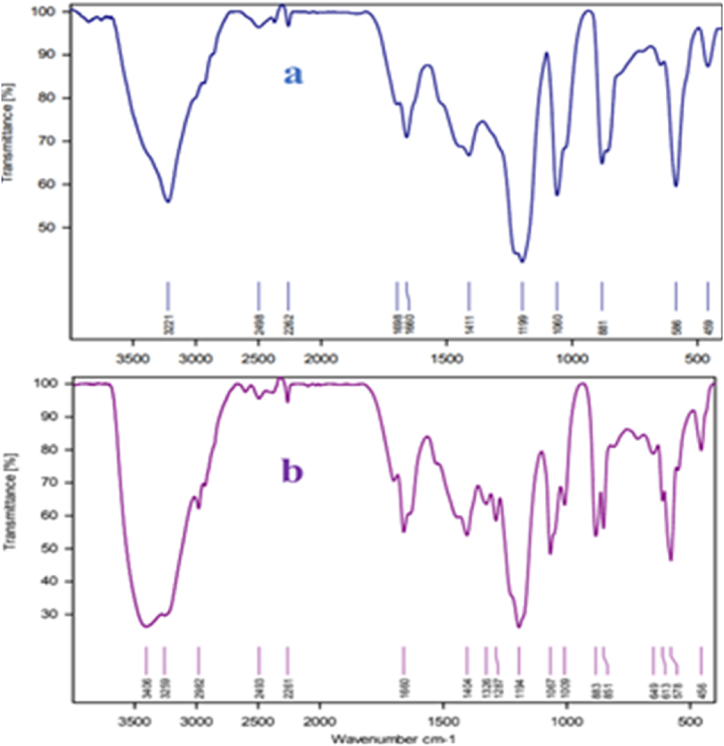
FT-IR spectrum of fresh of NA-NHSO_3_H (a) NA-NHSO_3_H after recovery (b).

**Fig. 15:**
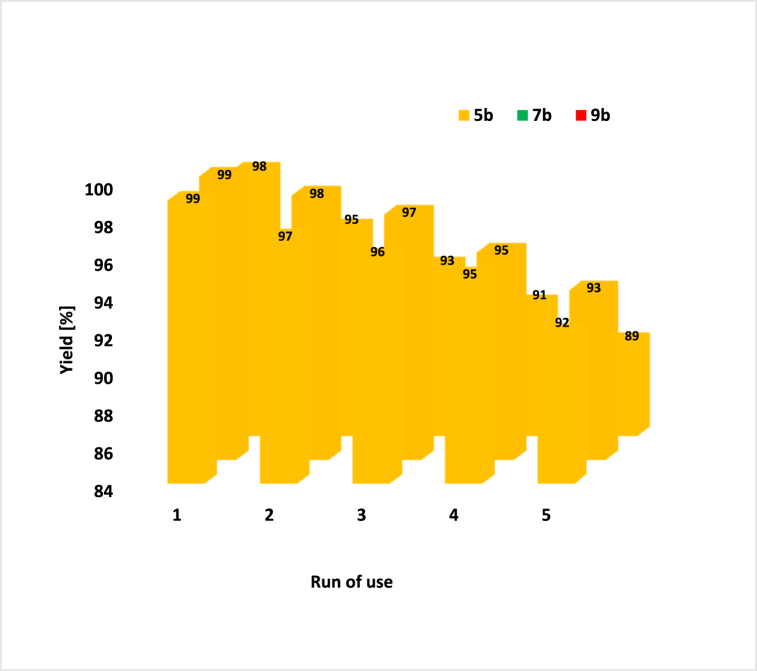
Recyclability of NA-NHSO_3_H in the synthesis of 5b, 7b and 9b.

SEM and FT-IR techniques were utilized to check the stability of the catalyst and its characteristics after reuse (Fig. 13, Fig. 14). The recycling of NA-NHSO_3_H was evaluated in 5b, 7b and 9b model reactions (Fig. 15). After each use, the catalyst was separated by filtration and the products were obtained by recrystallization in hot ethanol. It is clear that the catalyst activity can be used up to 5 times without a significant loss. Also, the histogram of the catalyst after recovery showed that the particle size average was 108 nm, which is completely consistent with the values obtained in the SEM and TEM histograms before it. Furthermore, the results indicated that the obtained spectra are in good agreement with the fresh catalyst and confirm that the shift of the peaks in FT-IR and the morphology of the spent catalyst does not have much effect even in the 5th cycle.

**Fig. 16:**
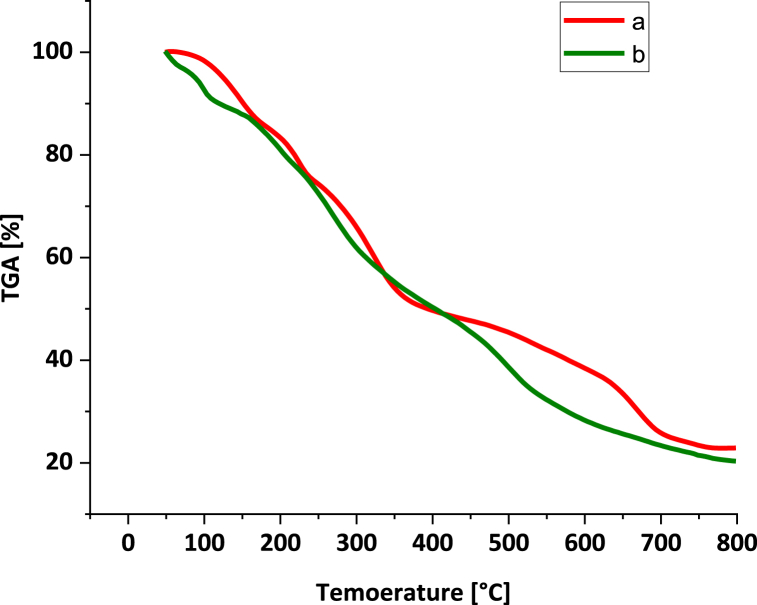
TGA of fresh of NA-NHSO_3_H (a) NA-NHSO_3_H after 5 runs (b).

TGA of fresh of NA-NHSO_3_H and NA-NHSO_3_H after 5 runs are shown in Fig. 16. The data from TGA provides compelling evidence that NA-NHSO_3_H remains thermally stable after multiple catalytic cycles. This finding supports its applicability as a reusable catalyst in various chemical reactions, paving the way for future research and industrial applications.

### 3.7 Hot filtration

The hot filtration test was performed to demonstrate the heterogeneous nature of the catalyst and to investigate the leaching of acidic sites. In this light, the reaction between 4-chlorobenzaldehyde, dimedone, ethyl acetoacetate and ammonium acetate at room temperature in 1 mL of H_2_O was selected as the model reaction. While running the experiment, the catalyst was separated in 10 min when the yield was 61 %, and the reaction mixture catalyst-free was stirred for another 10 min while maintaining the same conditions. At this stage, the product was obtained with a yield of 64 %. These results reveal that the stability of -SO_3_H groups on the natural asphalt support surface confirms the covalent adsorption of -SO_3_H on the catalytic support without acidic groups leaching into the solution or degrading it.

### 3.8 Comparison

A comparative study was applied for the use of NA-NHSO_3_H with some of the reported catalysts for the polyhydroquinoline, tetrahydrobenzo[b] pyran and 3,4-dihydropyrimidine-2(1H)-one reactions (Table 7). Reactions 3b, 4b and 5a were selected for comparison. Table 6 demonstrates that the catalytic system of our work has extensive advantages including mild reaction conditions, good to excellent and short reaction time. Thus, NA-NHSO_3_H showed better results than other catalysts, and in terms of green chemistry, it can be considered as one of the best choices for selecting a user-friendly benign and environmentally catalyst.

**Table 7:** Comparison of NA-NHSO_3_H ability in catalyzing of organic reactions with some reported catalyst.

**Entry**	**Catalyst (amount)**	**Product**	**Time (min)**	**Solvent**	**Temperature (°C)**	**Yield (%)**	**Ref.**
1	Urease (0.1 g)^a^	5b	210	H_2_O	65	86	[35]
2	magnetic dextrin (3.5 wt%)^a^	5b	15	EtOH	Reflux	95	[15]
3	Cu (ІІ)-PAA/M-MCM-41 (0.02 g)^a^	5b	20	Solvent-free	80	96	[36]
4	Fe_3_O_4_@chitosan (0.03 g)^a^	5b	70	EtOH	Reflux	90	[37]
5	NA-NHSO_3_H (10 mg)^a^	5b	15	H_2_O	r.t	99	This work
6	β -Cyclodextrin (2.0 mol %)^b^	7b	300	H_2_O	r.t	93	[38]
7	PFPA (35 mol%)^b^	7b	60	EtOH: H_2_O	r.t	92	[39]
8	SiO_2_-Pr-SO_3_H (0.03 g)^b^	7b	20	H_2_O	Reflux	90	[40]
9	HM-48 (20 wt%)^b^	7b	120	EtOH	60	80	[41]
10	NA-NHSO_3_H (10 mg)^b^	7b	15	H_2_O	r.t	99	This work
11	PPF-SO_3_H (0.18 g)^c^	9a	480	EtOH	Reflux	81	[8]
12	SBNPSA (0.05 g)^c^	9a	240	EtOH	Reflux	95	[6]
13	PS–PEG–SO_3_H (0.3 g)^c^	9a	600	Dioxane: 2-propanol (4:3)	80	86	[42]
14	TCCA (0.035 g)^c^	9a	720	EtOH	Reflux	94	[43]
15	bentonite/PS-SO_3_H (0.1 g)^c^	9a	30	Solvent-free	120	89	[44]
16	Cellulose sulfuric acid (0.05 g)^c^	9a	270	H_2_O	100	85	[10]
17	NA-NHSO_3_H (10 mg)^c^	9a	20	H_2_O	r.t	98	This work

a Reaction of 4-chlorobenzaldehyde, dimedone, ethyl acetoacetate and ammonium acetate for the synthesis of polyhydroquinoline.

b Reaction of 4-chlorobenzaldehyde, dimedone and malononitrile for the synthesis of tetrahydrobenzo[b] pyran.

c Reaction of benzaldehyde, ethyl acetoacetate and urea/thiourea for the synthesis of 3,4-dihydropyrimidine-2(1H)-one.

## 4 Conclusions

This study introduces NA-NHSO_3_H as a novel, active, and efficient Brønsted acid catalyst. Derived from natural asphalt (NA), this catalyst promotes the utilization of renewable resources and enhances the sustainability of heterocyclic compound synthesis. Its key advantages include ease of separation and reusability, making it a promising candidate for industrial applications. The NA-NHSO_3_H catalyst, prepared with green chemistry principles in mind, was characterized using various techniques. The research demonstrates a green and eco-friendly protocol for heterocyclic synthesis using -SO_3_H groups anchored within functionalized natural asphalt pores. This approach significantly improves catalytic activity. Its catalytic activity was successfully evaluated in the synthesis of polyhydroquinolines, tetrahydrobenzo[b]pyrans, and 3, 4-dihydropyrimidine-2(1H)-one/thione derivatives. The reactions were conducted with both electron-withdrawing and electron-donating aldehydes, yielding excellent product yields in all cases.

Key advantages of this method include: availability of natural materials, simple work-up procedure, use of green solvents, short reaction times, high yields, minimal waste generation and recyclable and reusable catalyst for up to 5 runs without significant yield loss.

Furthermore, this protocol enables the efficient and late-stage one-pot synthesis of various heterocyclic compounds on a gram scale. In conclusion, this innovative method represents a significant advancement in synthetic chemistry, promoting sustainability, efficiency, and high performance.

## CRediT authorship contribution statement

**Sahar Abdolahi:** Writing – original draft, Validation, Resources, Methodology, Conceptualization. **Mohammad Soleiman-Beigi:** Writing – review & editing, Supervision, Resources, Project administration.

## Data availability

All data generated or analysed during this study are included in this published article [and its supplementary information files].

## Declaration of competing interest

The authors declare the following financial interests/personal relationships which may be considered as potential competing interests: Mohammad Soleiman-Beigi reports financial support was provided by 10.13039/501100003968Iran National Science Foundation. If there are other authors, they declare that they have no known competing financial interests or personal relationships that could have appeared to influence the work reported in this paper.
