# Supplementary material for: Design of natural asphalt sulfamic acid (NA-NHSO3H) as a scalable natural asphalt-derived heterogeneous Brønsted acid to catalyze multicomponent reactions meeting green chemistry goals
Source: Heliyon. 2024 Dec 25;11(1):e41492. doi: 10.1016/j.heliyon.2024.e41492 (PMC11754174; doi:10.1016/j.heliyon.2024.e41492)
Supplement: Multimedia component 1 [file mmc1.docx]

**Supporting Information**

**Design of natural asphalt sulfamic acid (NA-NHSO_3_H) as a scalable natural asphalt-derived heterogeneous Brønsted acid to catalyze multicomponent reactions meeting green chemistry goals**

Sahar Abdolahi ^a^, Mohammad Soleiman-Beigi*^a^

^a^ *Department of Chemistry, Faculty of Basic Sciences, Ilam University, P.O. Box 69315516, Ilam, Iran.*

*E-mail:* [*SoleimanBeigi@yahoo.com*](mailto:SoleimanBeigi@yahoo.com)*;* [*m.soleimanbeigi@ilam.ac.ir*](mailto:m.soleimanbeigi@ilam.ac.ir)


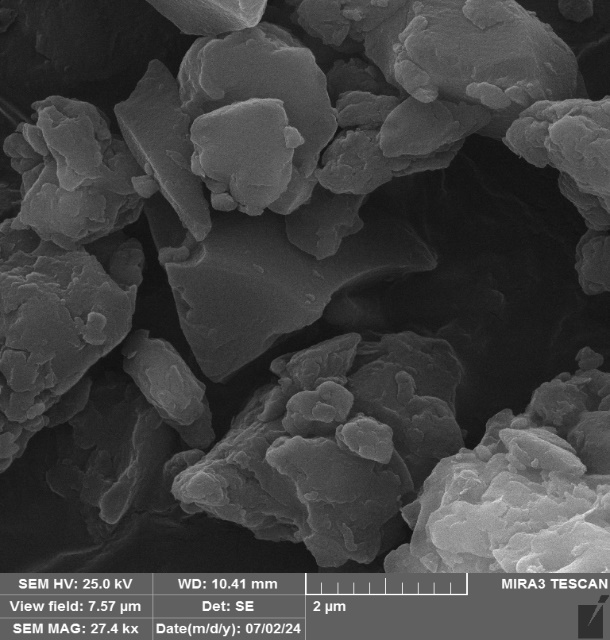

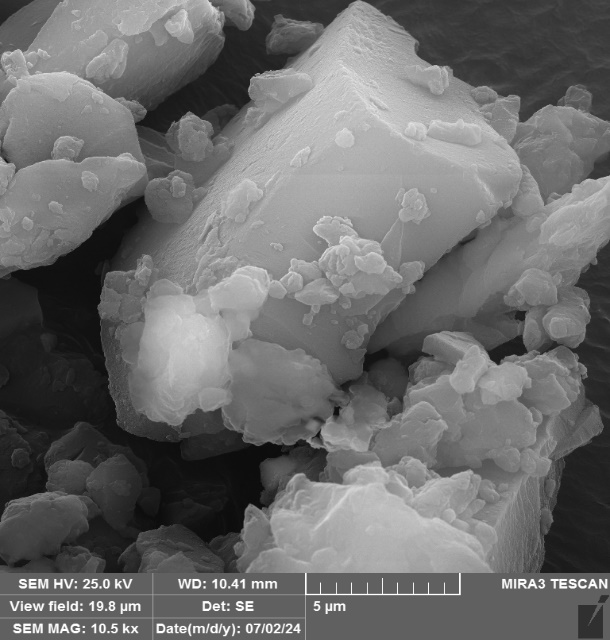

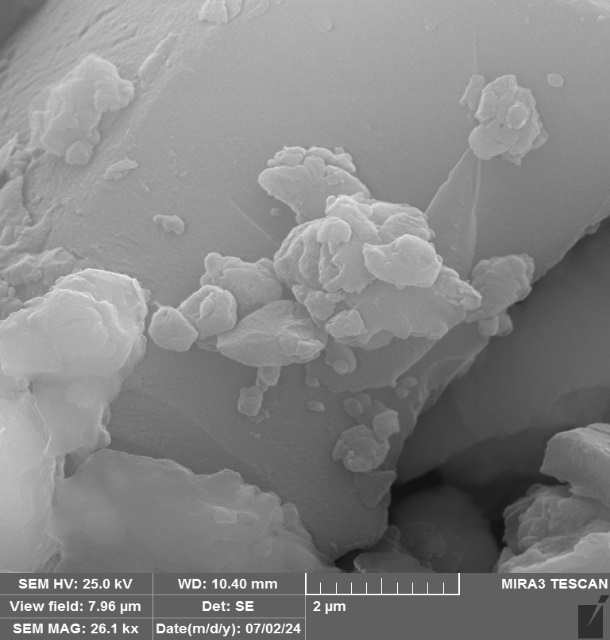

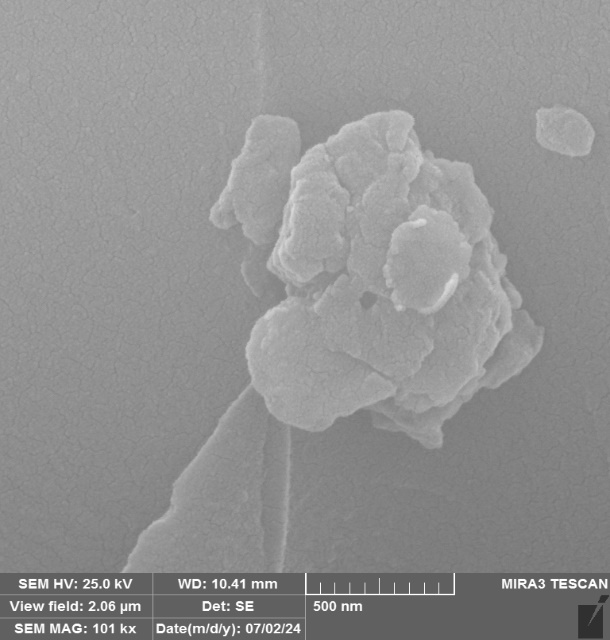


**Figure S1**: SEM images of NA


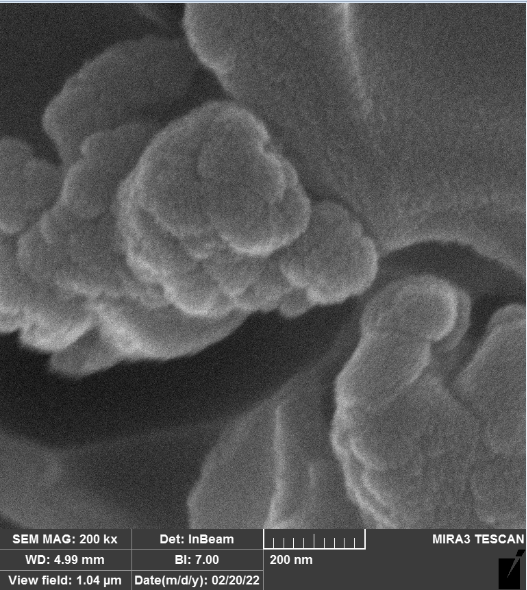

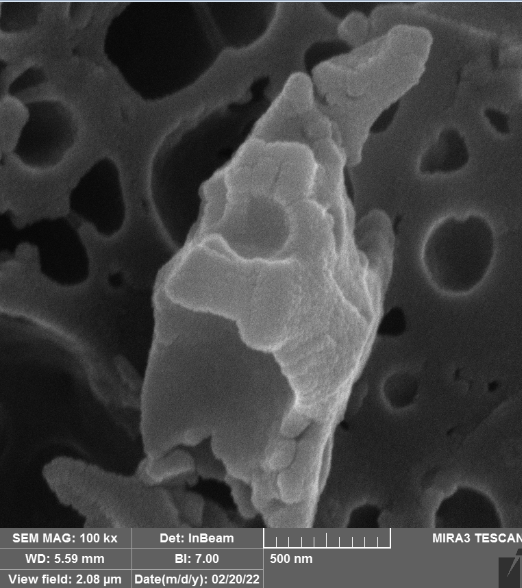

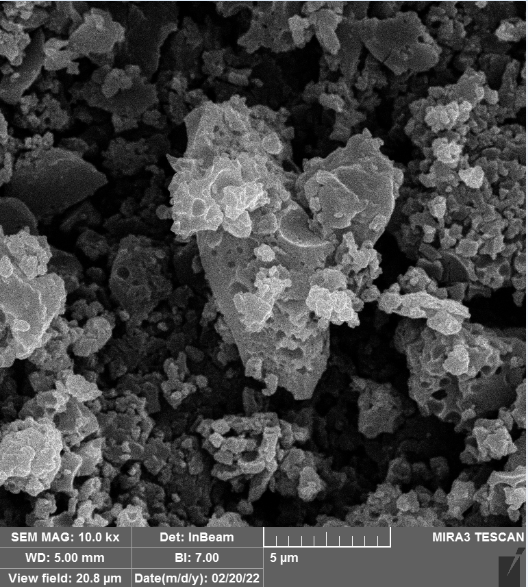

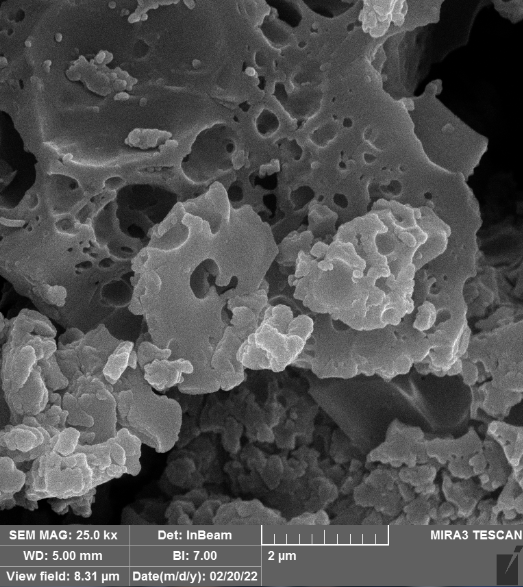


**Figure S2**: SEM images of NA-NO_2_


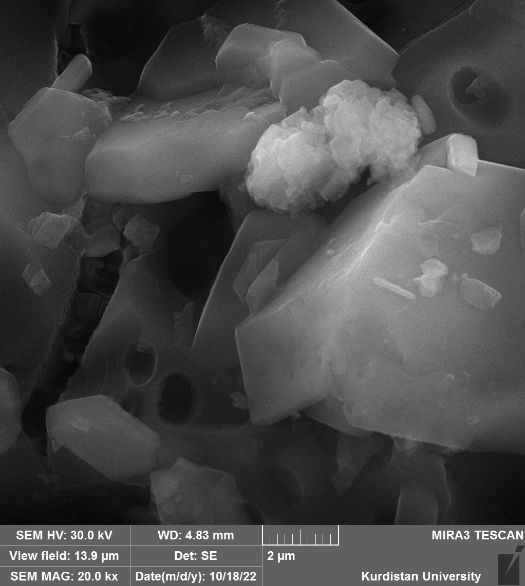

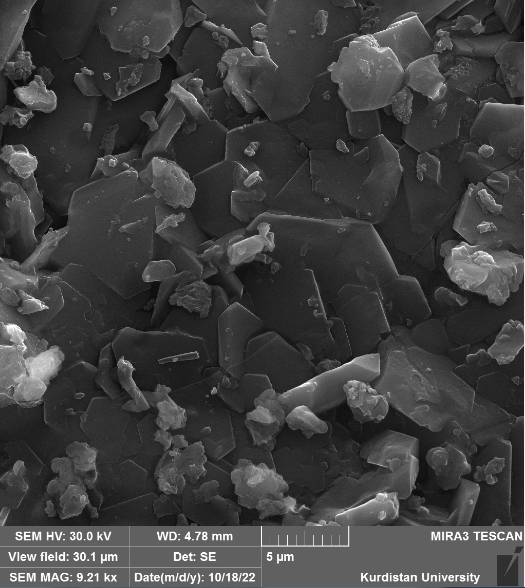

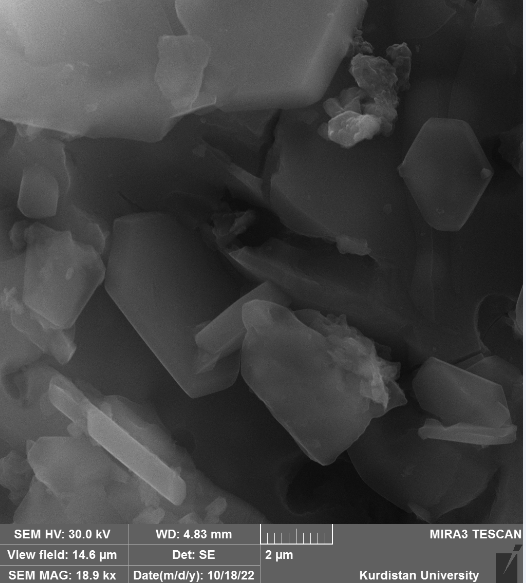

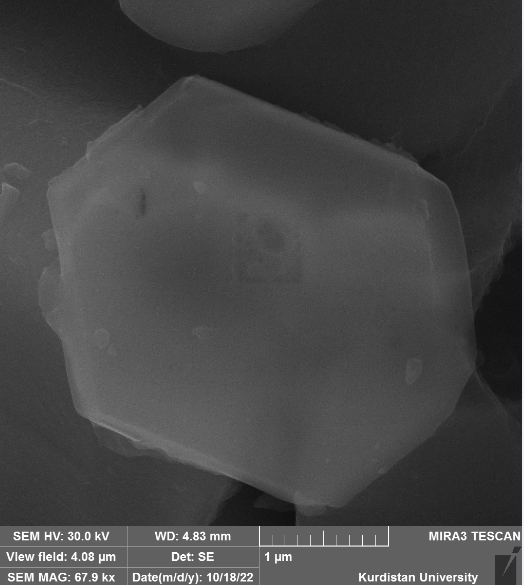


**Figure S3**: SEM images of NA-NH_2_


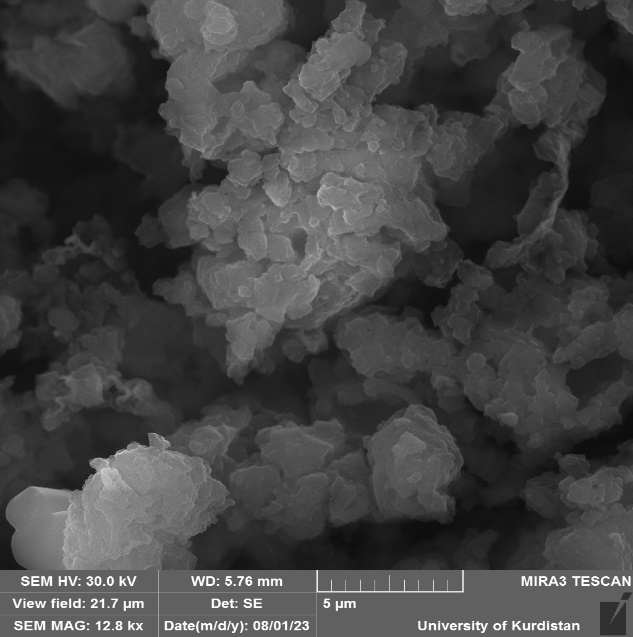

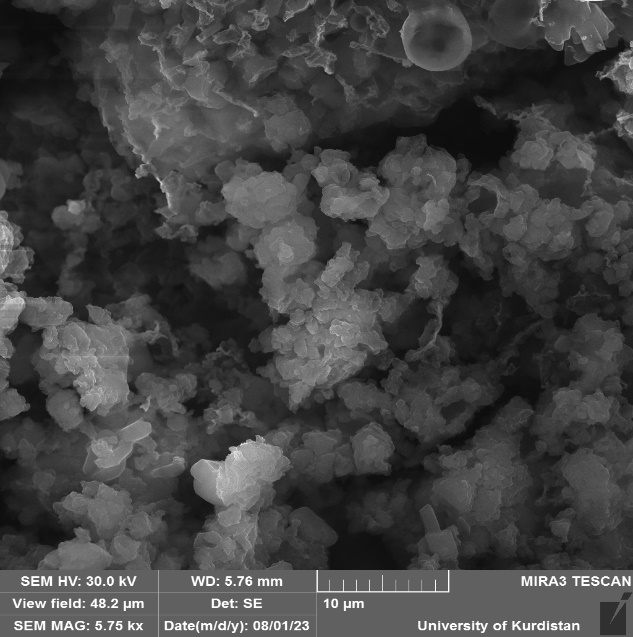

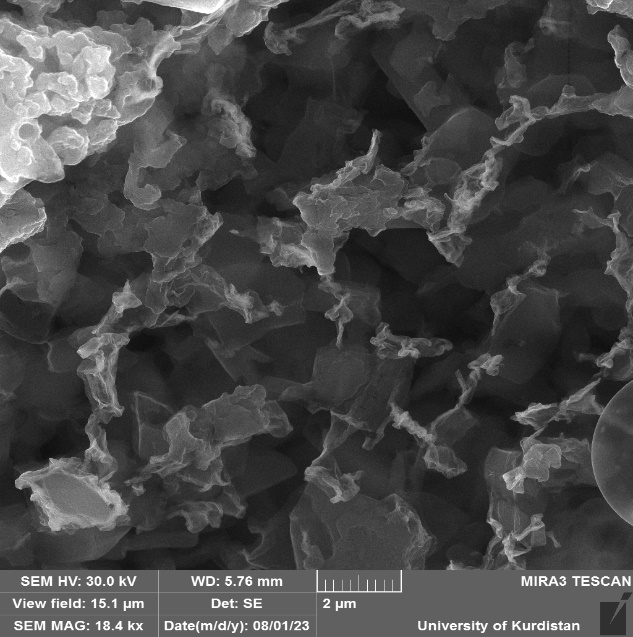

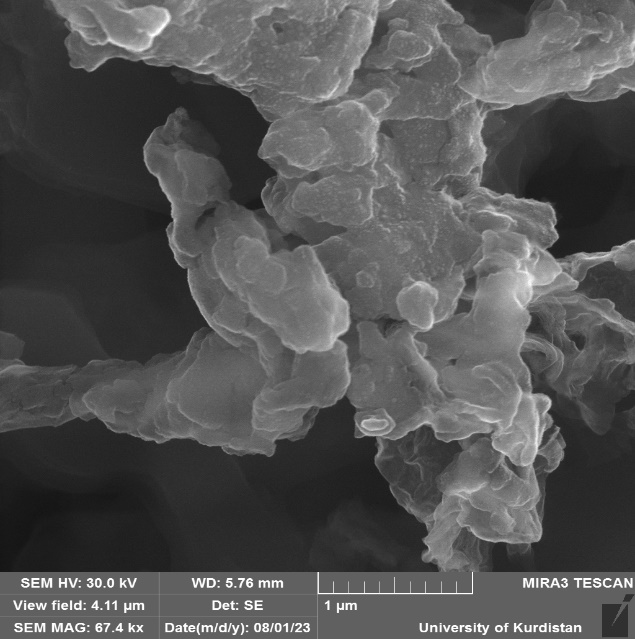


**Figure S4**: SEM images of NA-NHSO_3_H


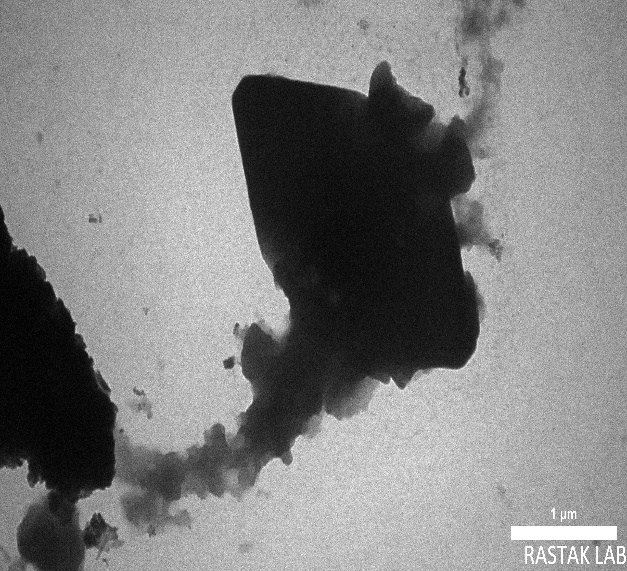

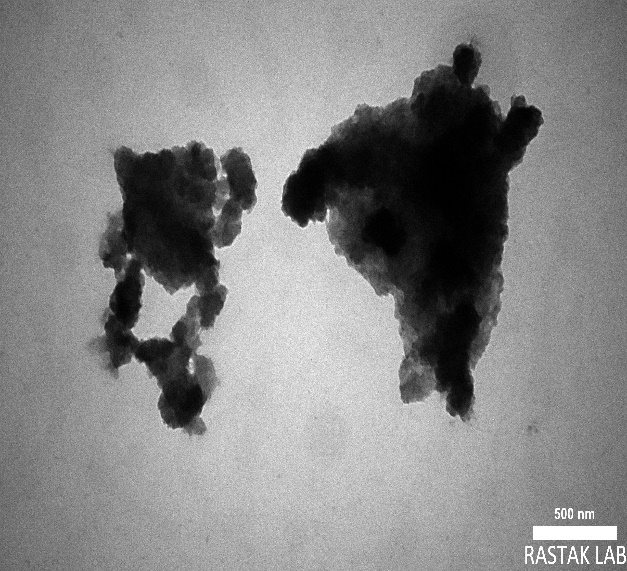

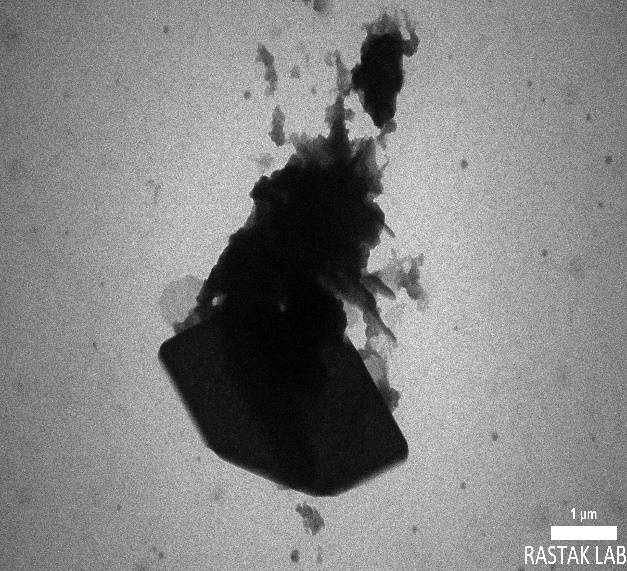

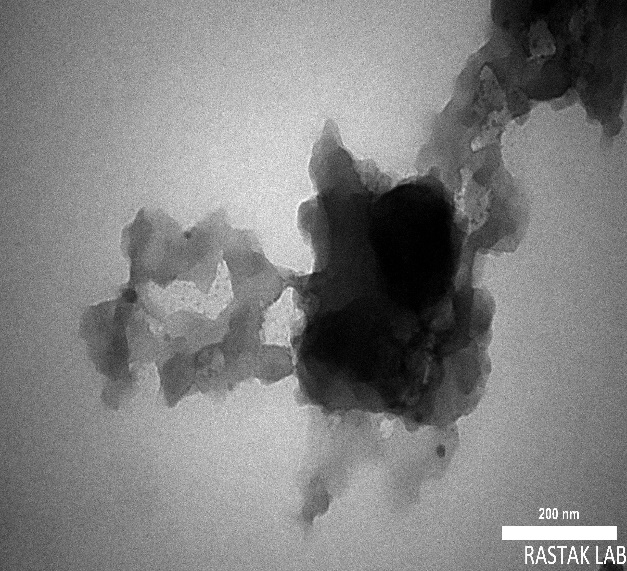


**Figure S5**: TEM images of NA-NHSO_3_H

Spectral data of some of the products:

***Ethyl‐4‐(4‐bromophenyl) ‐2,7,7‐ trimethyl‐5‐oxo‐1,4,5,6,7,8‐hexahydroquinoline‐3‐carboxylate (Figure S6, Table 4, 5d):*** Melting point: 257-260 °C. FT-IR (KBr) (v_max_/cm^-1^): 771, 1026, 1071, 1213, 1279, 1379, 1485, 1603, 1639, 1703, 2955, 3072, 3201, 3275 cm^-1^. ^1^H NMR (250 MHz, DMSO- d_6_): δ (ppm)= 9.14 (s, 1H, NH), 7.27 – 7.24 (d, *J* = 7.4 Hz, 2H), 7.16- 7.13 (d, *J* = 7.14 Hz, 2H), 4.80 (s, 1H), 3.99 – 3.94 (q, *J* = 3.96 Hz, 2H), 2.27 (s, 4H), 1.14- 1.08 (t, *J* = 1.11, 3H), 0.99 (s, 6H), 0.83 (s, 3H). ^13^C NMR (63 MHz, DMSO‐d_6_): δ (ppm)= 194.75, 167.03, 150.68, 150.33, 146.03, 130.79, 126.94, 121.43, 109.86, 103.43, 59.60, 50.58, 36.50, 32.61, 29.51, 26.82, 18.73, 14.53.

***2–Amino–3–cyano–7,7–dimethyl–4-(4-chlorophenyl)–5–oxo-4H–5,6,7,8-tetrahydro benzopyran (Figure S7, Table 5, 7b):*** Melting point: 210-212 °C. FT-IR (KBr) (v_max_/cm^-1^): 766, 1023, 1049, 1140, 1213, 1363, 1406, 1482, 1635, 1679, 2186, 3321, 3379 cm^-1^. ^1^H NMR (250 MHz, DMSO-*d*_6_): δ (ppm)= 7.32 (d, *J* = 8.1 Hz, 2H), 7.15 (d, *J* = 7.9 Hz, 2H), 7.02 (s, 2H), 4.17 (s, 1H), 2.23 (s, 4H), 1.01 (s, 3H), 0.92 (s, 3H). ^13^C NMR (63 MHz, DMSO-d_6_): δ (ppm)= 196.10, 163.05, 158.94, 144.16, 131.55, 129.55, 128.72, 119.97, 112.78, 58.26, 50.39, 35.56, 32.22, 28.74, 27.30.

***2–Amino–3–cyano–7,7–dimethyl–4-(******4-methylphenyl)–5–oxo-4H–5,6,7,8-tetrahydro benzopyran (Figure S8, Table 5, 7g):*** Melting point: 210-214 °C. FT-IR (KBr) (v_max_/cm^-1^): 760, 1025, 1142,1204, 1248, 1364, 1414, 1509, 1601, 1677, 2189, 3327, 3424 cm^-1^. ^1^H NMR (250 MHz, DMSO-*d*_6_): δ (ppm)= 7.04 (d, *J* = 9.2 Hz, 4H), 6.93 (s, 2H), 4.12 (s, 1H), 2.23 (s, 4H), 2.07 (s, 3H), 1.02 (s, 3H), 0.93 (s, 3H). ^13^C NMR (63 MHz, DMSO-d_6_): δ (ppm)= 196.03, 162.70, 158.89, 142.25, 136.05, 129.31, 127.51, 120.16, 113.34, 58.96, 50.46, 35.64, 32.22, 28.86, 27.22, 21.02.

***Ethyl-6-methyl-2-oxo-4-phenyl-1, 2, 3, 4-tetrahydropyrimidine-5-carboxylate (Figure S9, Table 6, 9a):*** Melting point: 202-204 °C. FT-IR (KBr) (v_max_/cm^-1^): 1039, 1223, 1291, 1313, 2925, 2979, 1462, 1647, 1725, 3116, 3245 cm^-1^. ^1^H NMR (250 MHz, DMSO-*d*_6_): δ (ppm)= 9.17 (s, 1H, NH), 7.72 (s, 1H, NH), 7.34 – 7.18 (m, 5H), 5.13 (s, 1H), 4.00 – 3.92 (q, 2H), 2.23 (s, 3H), 1.10 – 1.04 (t, 3H). ^13^C NMR (63 MHz, DMSO-d_6_): δ (ppm)= 160.09, 146.89, 143.08, 139.61, 123.13, 122.00, 120.99, 94.03, 53.92, 48.71, 12.51, 8.80.

***Ethyl-6-methyl-4-(2-hydroxyphenyl)-2-oxo-1,2,3,4-tetrahydropyrimidine-5-carboxylate (Figure S10, Table 6, 9i):*** Melting point: 200-202 °C. FT-IR (KBr) (v_max_/cm^-1^): 1088, 1233, 1271, 1329, 1371, 1464, 1501, 1599, 1675, 2906, 2942, 2990, 3348 cm^-1^.^1^H NMR (250 MHz, DMSO-*d*_6_): δ (ppm)= 9.55 (s, 1H), 9.07 (s, 1H), 7.06 (s, 1H), 7.03 – 6.93 (m, 2H), 6.79 – 6.93 (m, 2H), 5.45 (s, 1H), 3.96- 3.87 (q, J = 6.8 Hz, 2H), 2.26 (s, 3H), 0.99- 1.02 (t, J = 7.1 Hz, 3H). ^13^C NMR (63 MHz, DMSO-d_6_): δ (ppm)= 160.23, 149.41, 147.06, 143.31, 124.59, 123.01, 121.97, 113.46, 110.16, 92.55, 53.72, 43.90, 12.45, 8.76.


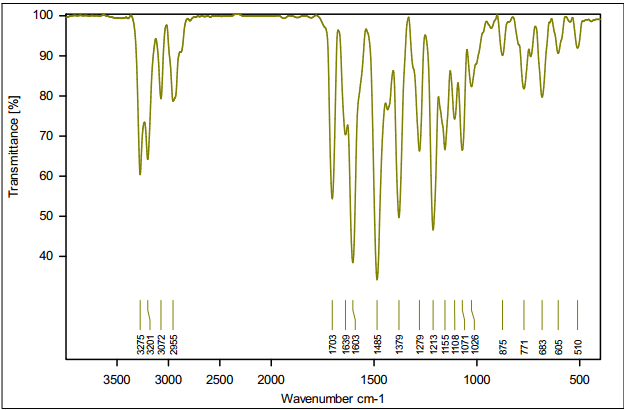


**(a)**


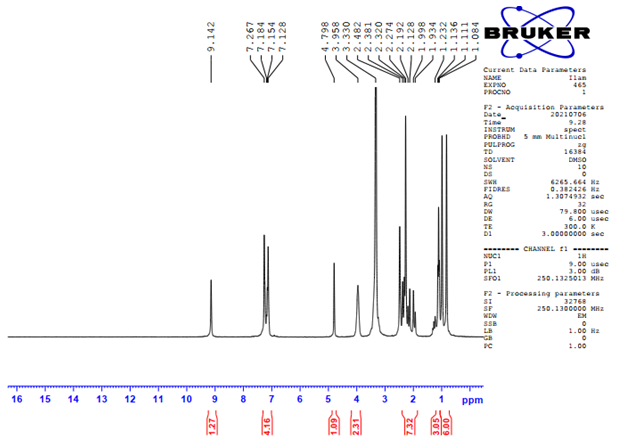


**(b)**


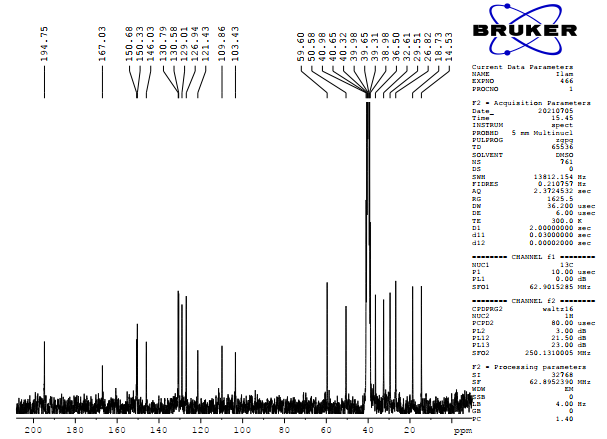


**(c)**

**Figure S6**: (a) FT-IR, (b) ^1^H-NMR and (c) ^13^C-NMR spectra of Ethyl‐4‐(4‐bromophenyl) ‐2,7,7‐ trimethyl‐5‐oxo‐1,4,5,6,7,8‐hexahydroquinoline‐3‐carboxylate.


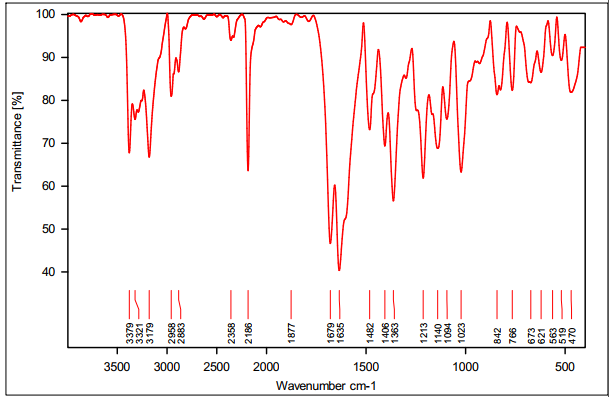


**(a)**


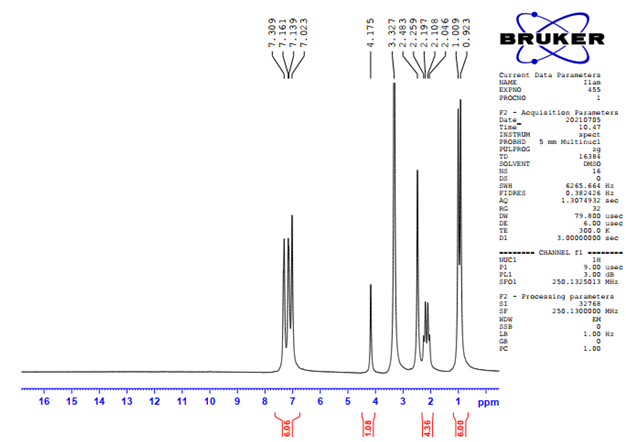


**(b)**


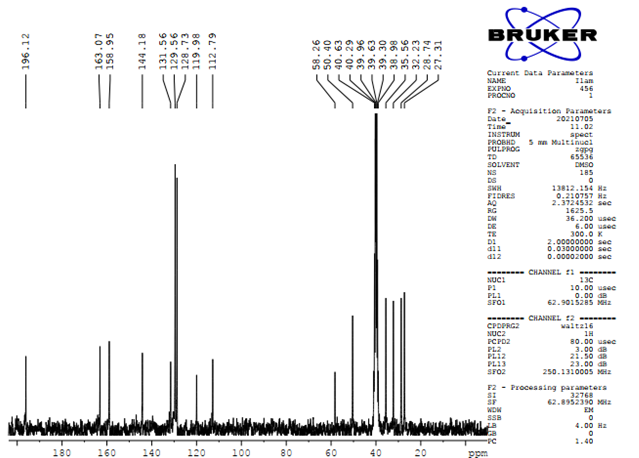


**(c)**

**Figure S7**: (a) FT-IR, (b) ^1^H-NMR and (c) ^13^C-NMR spectra of 2–Amino–3–cyano–7,7–dimethyl–4-(4-chlorophenyl)–5–oxo-4H–5,6,7,8-tetrahydro benzopyran.


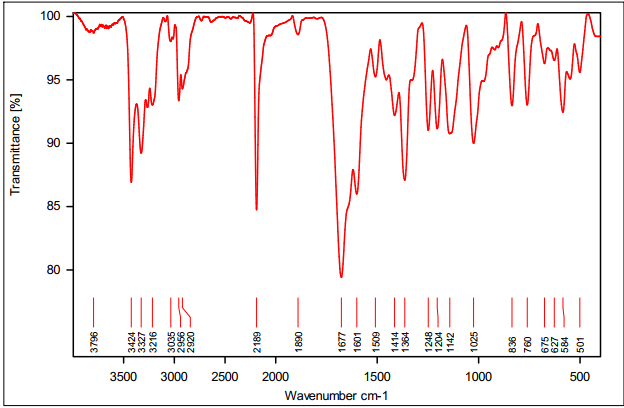


**(a)**


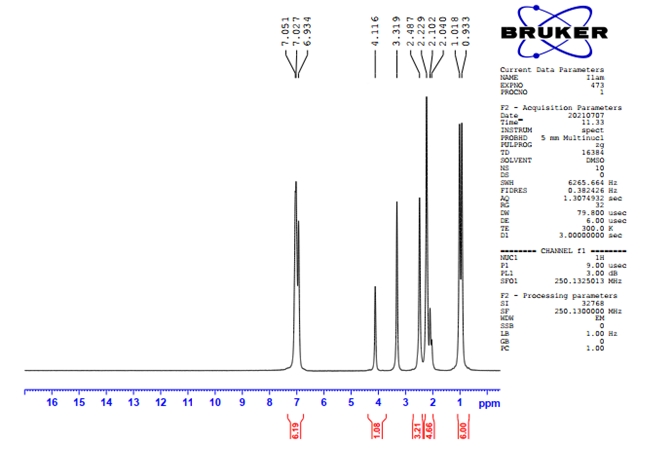


**(b)**


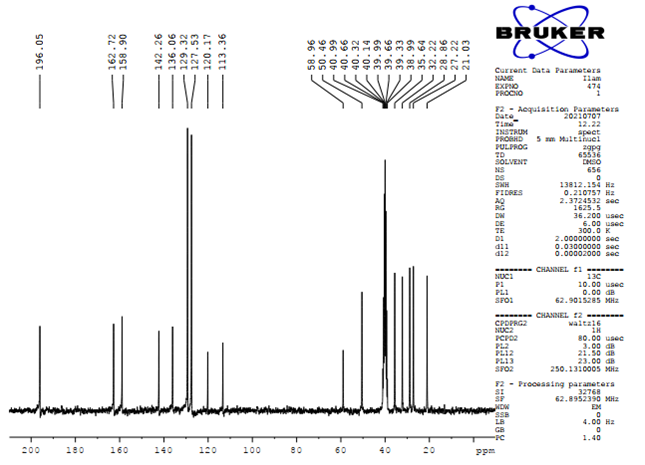


**(c)**

**Figure S8**: (a) FT-IR, (b) ^1^H-NMR and (c) ^13^C-NMR spectra of 2–Amino–3–cyano–7,7–dimethyl–4-(4-methylphenyl)–5–oxo-4H–5,6,7,8-tetrahydro benzopyran.


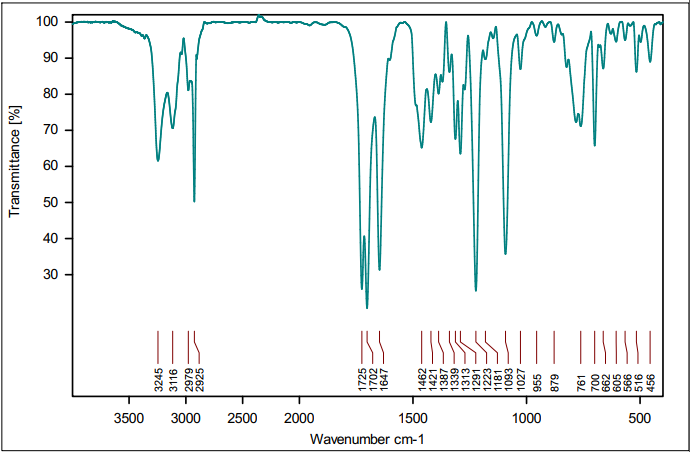


**(a)**


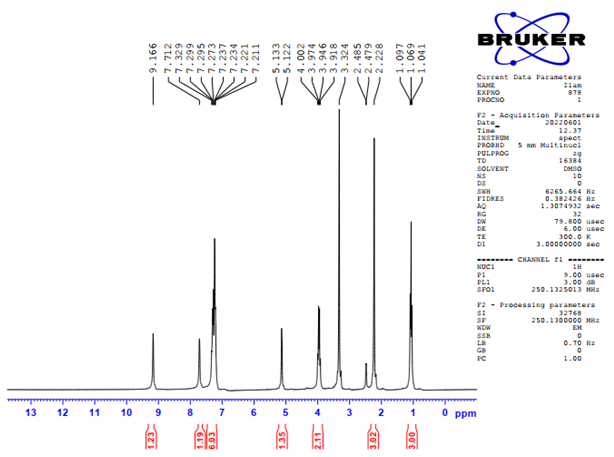


**(b)**


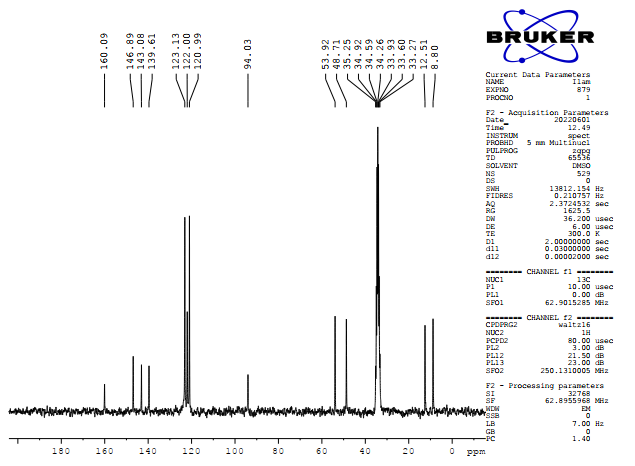


**(c)**

**Figure S9**: (a) FT-IR, (b) ^1^H-NMR and (c) ^13^C-NMR spectra of Ethyl-6-methyl-2-oxo-4-phenyl-1, 2, 3, 4-tetrahydropyrimidine-5-carboxylate.


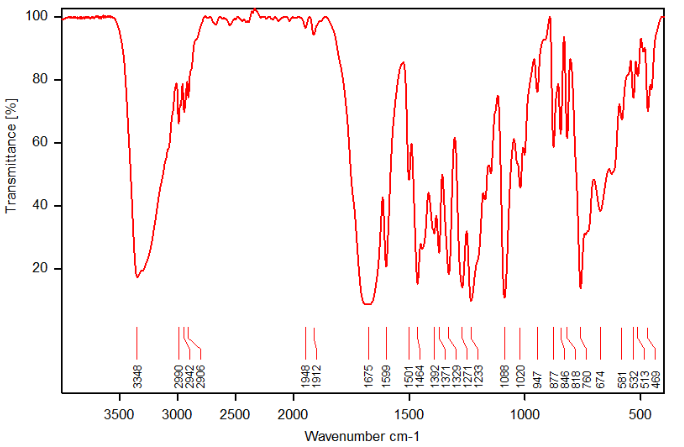


**(a)**


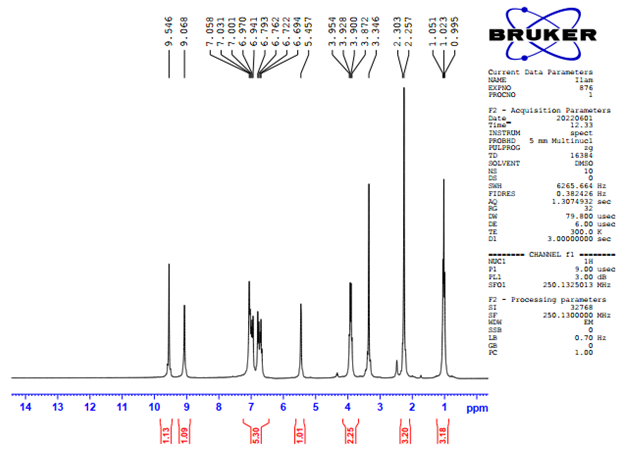


**(b)**


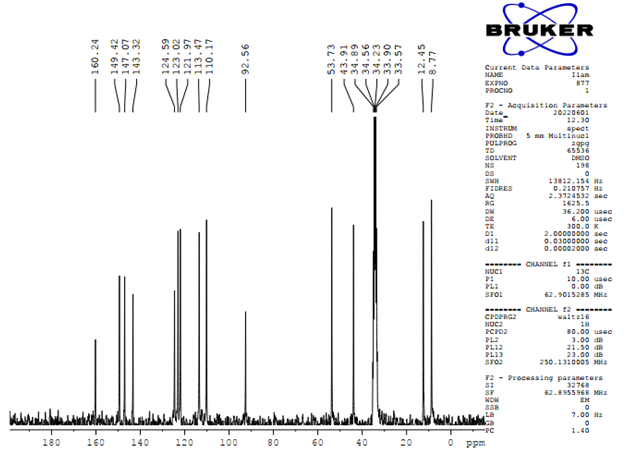


**(c)**

**Figure S10**: (a) FT-IR, (b) ^1^H-NMR and (c) ^13^C-NMR spectra of Ethyl-6-methyl-4-(2-hydroxyphenyl)-2-oxo-1,2,3,4-tetrahydropyrimidine-5-carboxylate.

**Synthesis of Ethyl‐4‐(4‐bromophenyl) ‐2,7,7‐ trimethyl‐5‐oxo‐1,4,5,6,7,8‐hexahydroquinoline‐3‐carboxylate (Table 4, 5d)**

**Experimental procedure:** 10 mg of NA-NHSO_3_H was added to a mixture of 4-bromorobenzaldehyde (1 mmol, 185 mg), dimedone (1 mmol, 140 mg), ethyl acetoacetate (1 mmol, 130 mg) and ammonium acetate (1.2 mmol, 93 mg) at room temperature in 1 mL of H_2_O. After the completion of the reaction by TLC, hot EtOH was added and NA-NHSO_3_H was separated by filtration. Finally, after evaporation of the solvent, the desired product was obtained and then purified through recrystallization in EtOH.

**Synthesis of 2–Amino–3–cyano–7,7–dimethyl–4-(4-chlorophenyl)–5–oxo-4H–5,6,7,8-tetrahydro benzopyran (Table 5, 7b)**

**Experimental procedure:** An molar ratio mixture of 4-chlorobenzaldehyde (1 mmol, 140 mg), dimedone (1 mmol, 140 mg) and malononitrile (1 mmol, 66 mg) in the presence NA-NHSO_3_H (10 mg) as a catalyst was reacted in H_2_O (1 mL) at room temperature. TLC was continuously applied during the completion of the reaction. After the starting material was consumed, hot EtOH was added and the catalyst was removed by filtration. Eventually recrystallization with EtOH was applied to afford the pure products.

**Synthesis of 2–Amino–3–cyano–7,7–dimethyl–4-(4-methylphenyl)–5–oxo-4H–5,6,7,8-tetrahydro benzopyran (Table 5, 7g)**

**Experimental procedure:** An molar ratio mixture of 4-methylbenzaldehyde (1 mmol, 120 mg), dimedone (1 mmol, 140 mg) and malononitrile (1 mmol, 66 mg) in the presence NA-NHSO_3_H (10 mg) as a catalyst was reacted in H_2_O (1 mL) at room temperature. TLC was continuously applied during the completion of the reaction. After the starting material was consumed, hot EtOH was added and the catalyst was removed by filtration. Eventually recrystallization with EtOH was applied to afford the pure products.

**Synthesis of Ethyl-6-methyl-2-oxo-4-phenyl-1, 2, 3, 4-tetrahydropyrimidine-5-carboxylate (Table 6, 9a)**

**Experimental procedure:** 10 mg of NA-NHSO_3_H was added to a mixture of benzaldehyde (1 mmol, 106 mg), ethyl acetoacetate (1 mmol, 130 mg) and urea (1.2 mmol, 72 mg) at room temperature in 1 mL of H_2_O. The reaction was monitored by TLC. After the completion of the reaction, hot EtOH was added and NA-NHSO_3_H was separated by filtration. Eventually, after evaporation of the solvent, the desired product was obtained and then purified through recrystallization in EtOH.

**Synthesis of Ethyl-6-methyl-4-(2-hydroxyphenyl)-2-oxo-1,2,3,4-tetrahydropyrimidine-5-carboxylate (Table 6, 9i)**

**Experimental procedure:** 10 mg of NA-NHSO_3_H was added to a mixture of 2-hydroxybenzaldehyde (1 mmol, 122 mg), ethyl acetoacetate (1 mmol, 130 mg) and urea (1.2 mmol, 72 mg) at room temperature in 1 mL of H_2_O. The reaction was monitored by TLC. After the completion of the reaction, hot EtOH was added and NA-NHSO_3_H was separated by filtration. Eventually, after evaporation of the solvent, the desired product was obtained and then purified through recrystallization in EtOH.
